# Tumor microenvironment-activated cancer cell membrane-liposome hybrid nanoparticle-mediated synergistic metabolic therapy and chemotherapy for non-small cell lung cancer

**DOI:** 10.1186/s12951-021-01085-y

**Published:** 2021-10-24

**Authors:** Wei Zhang, Chunai Gong, Ziqiang Chen, Ming Li, Yuping Li, Jing Gao

**Affiliations:** 1 Department of Pharmacy, Shanghai Pulmonary Hospital, Tongji University School of Medicine, Shanghai, 200433 China; 2 Department of Pharmacy, Shanghai Ninth People’s Hospital, Shanghai JiaoTong University School of Medicine, Shanghai, 201999 China; 3 Department of Orthopaedic, Shanghai Changhai Hospital, Naval Medical University, Shanghai, 200433 China; 4 State Key Laboratory of Toxicology and Medical Countermeasures, Beijing Institute of Pharmacology and Toxicology, Beijing, 100850 China

**Keywords:** Biomimetic nanoparticles, Hybrid nanovesicle, Tumor microenvironment activated, Glycolysis, Chemotherapy, Non-small cell lung cancer

## Abstract

**Background:**

Biomimetic nanotechnology-based RNA interference (RNAi) has been successful in improving theranostic efficacy in malignant tumors. Its integration with hybrid biomimetic membranes made of natural cell membranes fused with liposomal membranes is mutually beneficial and extends their biofunctions. However, limited research has focused on engineering such biomimetics to endow them with unique properties and functions, in particular, those essential for a “smart” drug delivery system, such as a tumor microenvironment (TME)-activated multifunctional biomimetic nanoplatform.

**Results:**

Herein, we utilized an integrated hybrid nanovesicle composed of cancer cell membranes (Cm) and matrix metallopeptidase 9 (MMP-9)-switchable peptide-based charge-reversal liposome membranes (Lipm) to coat lipoic acid-modified polypeptides (LC) co-loaded with phosphoglycerate mutase 1 (*PGAM1*) siRNA (siPGAM1) and DTX. The nanovesicle presented a negatively charged coating (citraconic anhydride-grafted poly-l-lysine, PC) in the middle layer for pH-triggered charge conversion functionalization. The established chemotherapeutic drug (DTX) co-delivery system CLip-PC@CO-LC nanoparticles (NPs) have a particle size of ~ 193 nm and present the same surface proteins as the Cm. Confocal microscopy and flow cytometry results indicated a greater uptake of MMP-9-treated CLip-PC@CO-LC NPs compared with that of the CLip-PC@CO-LC NPs without MMP-9 pretreatment. The exposure to MMP-9 activated positively charged cell-penetrating peptides on the surface of the hybrid nanovesicles. Moreover, pH triggered membrane disruption, and redox triggered DTX and siRNA release, leading to highly potent target-gene silencing in glycolysis and chemotherapy with enhanced antiproliferation ability. The biodistribution results demonstrated that the CLip-PC@LC-DiR NPs accumulated in the tumor owing to a combination of long blood retention time, homologous targeting ability, and TME-activated characteristics. The CLip-PC@CO-LC NPs led to more effective tumor growth inhibition than the DTX and free siPGAM1 formulations.

**Conclusions:**

TME-activated cancer cell membrane-liposome integrated hybrid NPs provide an encouraging nanoplatform that combines RNAi with chemotherapy for precise treatment of non-small cell lung cancer.

**Graphical abstract:**

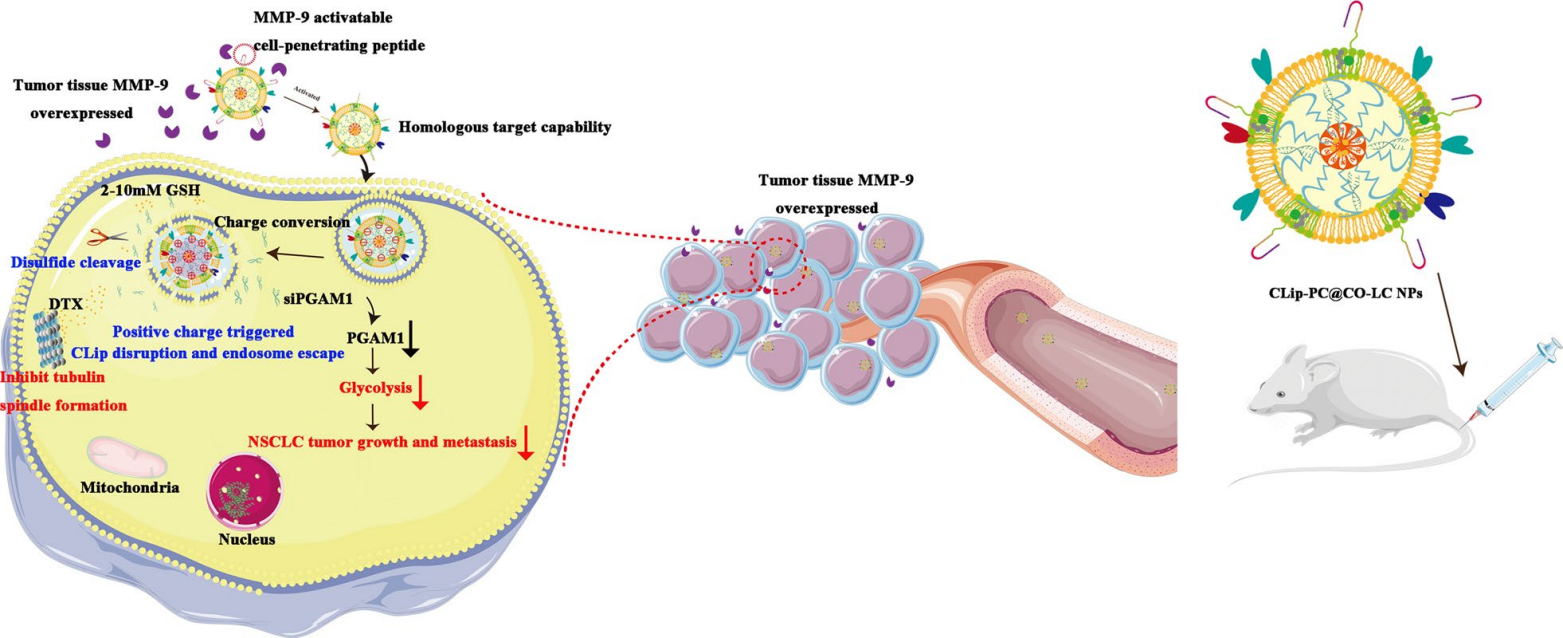

**Supplementary Information:**

The online version contains supplementary material available at 10.1186/s12951-021-01085-y.

## Background

Lung cancer is one of the commonest and most devastating human malignancies, accounting for approximately one-quarter of all cancer deaths, and has a 5-year survival rate of 20.5% [1, 2]. The major pathological subtype of lung cancer is non-small cell lung cancer (NSCLC), which comprises 85% of all cases [3]. Most NSCLC cases are diagnosed at advanced stages, which is the main reason for poor prognosis [4]. Epidermal growth factor receptor tyrosine kinase inhibitors (EGFR-TKIs) are the first-line treatment for patients with lung adenocarcinoma with sensitizing EGFR mutations [5, 6]. Although EGFR-TKIs are initially effective, patients inevitably develop resistance [7, 8]. At present, taxol is the most effective anticancer agent in first-line management for patients with NSCLC [5]. Despite improvements in disease control and survival with current chemotherapeutic regimens, challenges remain in clinical therapy due to resistance to chemotherapeutic agents and the invasiveness and metastasis of NSCLC, which result in treatment failure and ultimately, death [9–11]. Therefore, the development of safe and effective strategies against lung cancer is urgently required.

Metabolic aberrance, especially aerobic glycolysis, is one of the primary hallmarks of malignancy and supports proliferation, invasion, metastasis, and resistance to cancer treatments [12, 13]. It was first reported by Warburg and is often referred to as the Warburg effect [14]. Phosphoglycerate mutase 1 (PGAM1), a key aerobic glycolysis enzyme in cancer metabolism, dynamically converts 3-phosphoglycerate (3-PG) to 2-phosphoglycerate (2-PG), which regulates glycolysis, pentose phosphate pathway (PPP) flux, and biosynthesis [15]. PGAM1 is frequently upregulated in multiple malignancies, including lung cancer, hepatocellular carcinoma, and colorectal cancer [16–18]. Elevated levels of PGAM1 are correlated with poor prognosis for NSCLC patients, and knockdown of *PGAM1* suppresses aggressive cancer phenotypes as well as mTOR-mediated glycolysis and oncogenesis in NSCLC cells [19, 20]. Recently, the combination of glycolytic inhibitors with chemotherapy as a promising strategy to achieve synergistic treatment in highly glycolytic tumors has been suggested [21–23].

RNA interference (RNAi) is a naturally occurring mechanism for gene therapy since its mammalian gene function interference effect was first reported in 2001 [24]. However, because of its inherent vulnerability in biological environments, suboptimal knockdown efficiency, and low bio-membrane permeability, efficient and targeted delivery is one of the greatest challenges in reaching its full potential [25, 26]. Nano-based systems-mediated siRNA delivery is an effective approach for improving bioavailability and biocompatibility [27]. Yet, therapeutic siRNA-based down-regulated gene expression in solid tumors remains an obstacle for the application of RNAi in cancer therapy, which requires efficient delivery technology to transport siRNA into the cytosol [28].

**Scheme 1:**
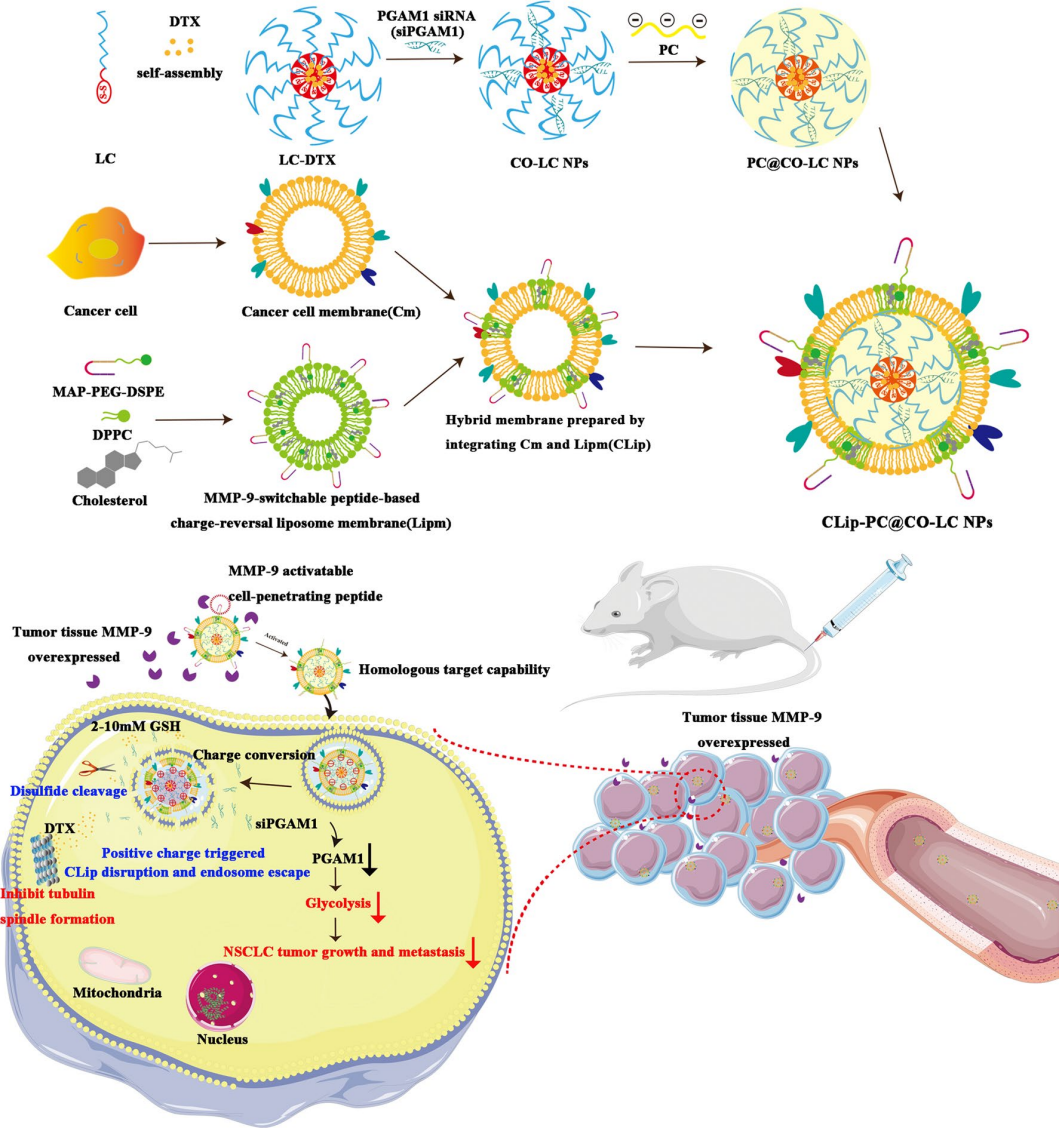
A schematic diagram of the synthesis of CLip-PC@CO-LC NPs and the intracellular uptake

We have previously identified, through the imaginative use of lipoic acid-modified polypeptide (LC) micellar system, the main composition of a cell-penetrating peptide (CPP) that can mediate the non-endosomal mechanism of cytoplasmic siRNA delivery, which could effectively co-deliver siRNA and chemotherapeutic drugs to target cells [29]. In this study, we have extended the application of LC to antitumor treatment and developed a tumor microenvironment (TME)-activated biomimetic nanoparticle (NP)-based nanosystem (CLip-PC@CO-LC NPs) using an LC micelle, which was used for the co-delivery of DTX and *PGAM1* siRNA (siPGAM1). A cancer cell membrane (Cm) can be used to endow final NPs with homologous targeting ability, improve the efficiency of drug delivery to the target cells, and enhance therapeutic efficiency [30, 31]. Moreover, compared with mono-membrane coating, liposome membranes are more easily modified and can be integrated into a single biomimetic platform to obtain diverse functionalities for the precise treatment of cancer [32, 33]. Hence, we constructed an integrated hybrid nanovesicle, cancer cell membrane lipsome (CLip), by fusing Cm and MMP-9-switchable peptide-based charge-reversal liposome membrane (Lipm), thereby utilizing both biomaterials. MMP-9 is a protease upregulated in various malignant tumors [34, 35], including NSCLC [36, 37]. An MMP-9-sensitive peptide (RRRRRRRRR-PVGLIG-EGGEGGEGG) was integrated into the lipid membrane of the liposomes. This shows a negative surface charge in the systemic circulation, exhibiting advantageous stability, accumulating at the tumor sites where MMP-9 is overexpressed, exposing the positively charged CPP following MMP cleavage, and resulting in enhanced internalization into target cells [38]. Citraconic anhydride-grafted poly-l-lysine (PC) was used as the negatively charged coating in the middle layer for pH-triggered charge conversion; hydrolysis of citraconic amide under a mildly acidic TME could exhibit reversible negative-to-positive charge transition. The charge-reversal core would lead to the collapse and disruption of the chemotherapeutic drug DTX co-delivery system, which could be conducive to efficient siRNA delivery [39, 40]. Systematic evaluation of the resulting TME-activated biomimetic siPGAM1 and the CLip-PC@CO-LC NPs (Scheme 1), both in vitro and in vivo, was conducted. We present this multifunctional biomimetic nanocarrier as proof of concept for an innovative integrative strategy for efficient siRNA delivery, and introduce a new generation of biomimetic nanovehicles with stimuli responsiveness for enhanced drug co-delivery.

## Results and discussion

### Preparation and characterization of fusogenic CLips

**Fig. 1:**
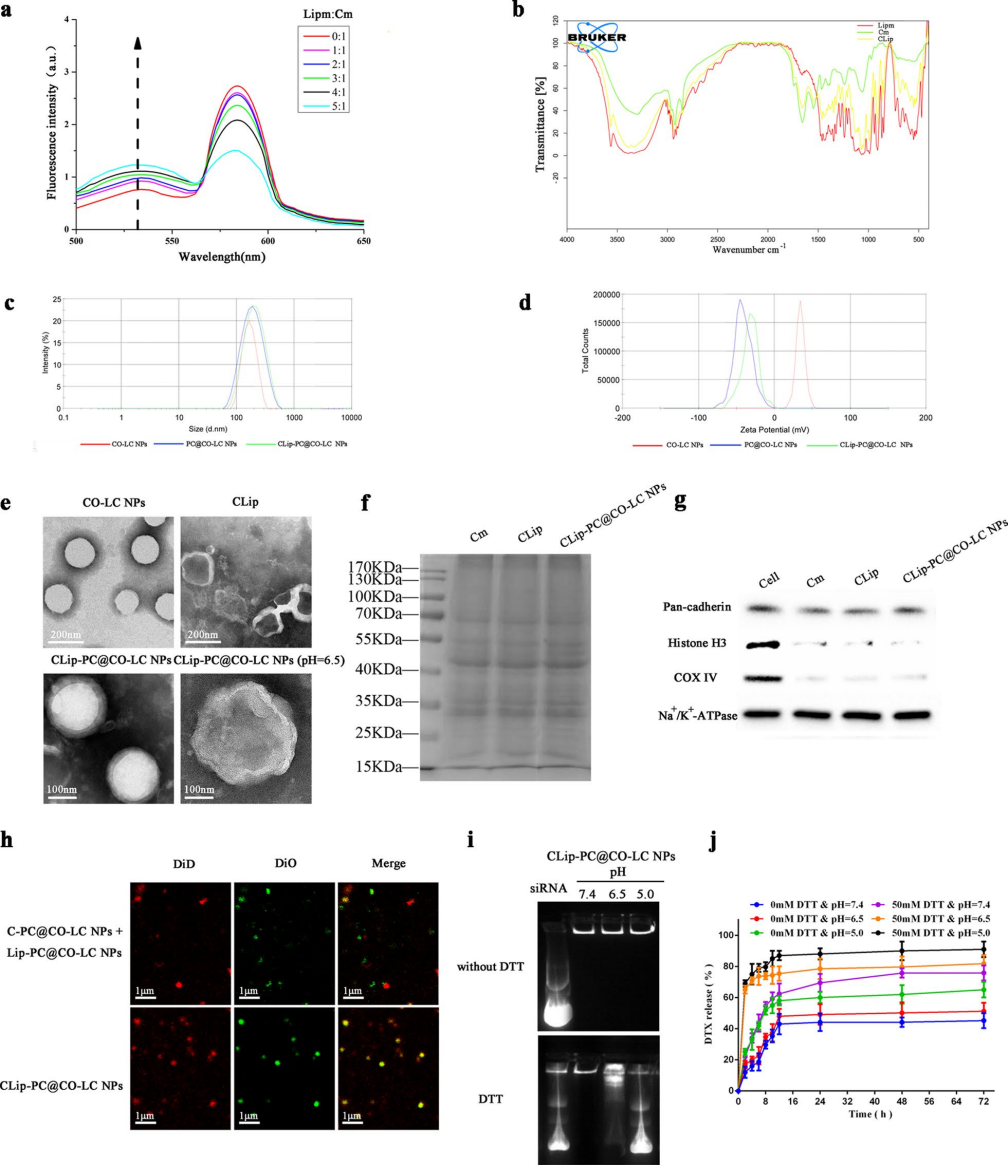
Characterization of CLip-PC@CO-LC nanoparticles (NPs). **a** A549 cell membrane was labeled with DOPE-RhB and C6-NBD as a fluorescence resonance energy transfer (FRET) pair of dyes and fused with an increasing number of liposomes; their fluorescence spectra were then detected and recorded. (Lipm:Cm represents the weight ratio of liposomes to A549 cell membrane proteins). **b** The Fourier transform infrared (FTIR) spectra of Lipm, Cm, and CLip verified the retention of A549 cell membrane proteins in CLips. **c** Average hydrodynamic size of CO-LC NPs, PC@CO-LC NPs, and CLip-PC@CO-LC NPs (n = 3). **d** Zeta potential of CO-LC NPs, PC@CO-LC NPs, and CLip-PC@CO-LC NPs (n = 3). **e** Representative transmission electron microscopy (TEM) images of CO-LC NPs, CLip, CLip-PC@CO-LC NPs, and CLip-PC@CO-LC NPs (pH = 6.5) negatively stained with phosphotungstic acid (scale bars = 200 nm and 100 nm). **f** Profiles of proteins in Cm, CLip, and CLip-PC@CO-LC NPs determined using SDS-PAGE. **g** Western blot analysis of pan-cadherin, histone H3, and COX IV in A549 cells, Cm, CLip, and CLip-PC@CO-LC NPs. **h** Confocal fluorescent microscopy images of CLip-PC@CO-LC NPs and the mixture of C-PC@CO-LC NPs, Lip-PC@CO-LC NPs (red = Cm, green = liposomes; scale bar = 1 µm). **i** Electrophoretic gel assays of CLip-PC@CO-LC NPs in the presence or absence of DTT at different pH values (pH = 7.4, 6.0, and 5.0). **j** DTX release from CLip-PC@CO-LC NPs in the presence or absence of DTT at different pH values (pH = 7.4, 6.5, and 5.0)

CLips were prepared by fusing an A549 Cm with Lipm, as illustrated in Scheme 1. We assayed the protein content of Cm by using a bicinchoninic acid (BCA) assay kit. The average protein content in the A549 cell membrane extracted from 1 × 10^8^ cells was found to be 0.65 ± 0.12 mg. For Lipm preparation, we prepared fusogenic liposomes using the film hydration technique. The membrane fusogenic properties of Cm and Lipm were evaluated using fluorescence resonance energy transfer (FRET) assay. The Cm was labeled with DOPE-RhB and C6-NBD as a pair of FRET dyes and then added to different amounts of Lipm. As shown in Fig. 1a, a recovery of fluorescence at 534 nm and a reduction in fluorescence at 583 nm with an increasing amount of Lipm were observed, suggesting interluding of the lipid and cell membrane materials and attenuation of the FRET of the dyes. In addition, the hybridization of Lipm and Cm in CLip was observed using Fourier transform infrared (FTIR) spectroscopy to verify the functional characteristics of the membrane proteins. Figure 1b shows similar typical protein absorption bands of tumor cell membrane proteins present in the Cm and CLip groups, indicating the fusion of the two membrane materials. Among the absorption bands, 1700^–1^, 600 cm^−1^ and 1600^–1^, 500 cm^−1^ band regions indicated NH bending with C–N and C=O stretching vibrations, respectively. Combined, these results strongly confirmed the perfect formation of Cm and Lipm as well as the retention in CLip of membrane proteins inherited from the Cm.

### Physicochemical characterization of CLip-PC@CO-LC NPs

PC was used as a negative coating in the middle layer of the biomimetic nanocomplex, which exhibited pH-triggered charge-conversion. ^1^H NMR spectroscopy was conducted to determine the structural characteristics of PC. As shown in Additional file 1: Figure S1, the characteristic peaks of methylene groups (peaks e–g) from the lysine units appeared at 1.06–1.16 ppm; the characteristic peaks of methenyl groups from the lysine units appeared at 4.06 ppm (peak b). A peak of δc 3.02 ppm corresponded to δc (–CH_2_–NH–CO–). Small peaks, which were assigned to methyl protons from citraconic anhydride units, at 5.72 ppm (peak a) and 1.94 ppm (peak d) confirmed the successful conjugation of citraconic anhydride to poly-l-lysine.

DTX and siPGAM1 co-loaded polypeptide micelles were fabricated using the procedure described in our previous study [29]. CO-LC NPs were coated with PC by incubation to prepare PC@CO-LC NP nanocomposites [39]. Then, CLip was extracted and sonicated with PC@CO-LC NPs according to a previously reported method [41] and was used to further prepare the CLip-PC@CO-LC NPs. Dynamic light scattering (DLS) showed that the size of CLip-PC@CO-LC NPs was approximately 193.6 ± 0.97 nm (polydispersity index, PDI, 0.20 ± 0.06), while those of CO-LC NPs and PC@CO-LC NPs were 160.4 ± 1.86 nm (PDI, 0.09 ± 0.03) and 172.8 ± 1.92 nm (PDI, 0.12 ± 0.02), respectively (Fig. 1c). The zeta potential of the CO-LC NPs was 35.4 ± 1.18 mV, whereas the zeta potential of the prepared PC@CO-LC NPs and CLip-PC@CO-LC NPs was − 41.2 ± 0.70 mV and − 33.5 ± 1.18 mV, respectively. Compared to the positive zeta potential of CO-LC NPs, the charge of PC@CO-LC NPs became negative, which further verified the successful coating of PC on CO-LC NPs (Fig. 1d). The responsiveness of the CLip-PC@CO-LC NPs was confirmed in a simulated biological environment. Compared to the size of CLip-PC@CO-LC NPs at pH 7.4 (195.2 ± 2.47 nm), the diameter increased sharply at pH 6.5 (368.9 ± 4.75 nm) and appeared to have larger hydrodynamic sizes at pH 5.0 (546.2 ± 6.62 nm) (Additional file 1: Figure S2a). In addition, as shown in Additional file 1: Figure S2b, compared to the zeta potential of CLip-PC@CO-LC NPs at pH 7.4 (− 33.5 ± 0.86 mV), the surface zeta potential of CLip-PC@CO-LC NPs increased significantly at pH 6.5 (8.38 ± 0.54 mV) and pH 5.0 (17.3 ± 1.70 mV), indicating the successful charge conversion of PC as the pH decreased. The negligible change in size in the presence or absence of MMP-9 illustrated suitable stability of CLip-PC@CO-LC NPs in vitro (Additional file 1: Figure S2c). As shown in Additional file 1: Figure S2d, CLip-PC@CO-LC NPs (without MMP-9) were negatively charged, with a zeta potential of − 32.03 ± 1.63 mV. After treatment with MMP-9, MMP-9-sensitive CPP was cleaved to expose the inner positively charged cell-penetrating sequence. CLip-PC@CO-LC NPs (with MMP-9) further cleaved the MMP-9-sensitive peptide sequence to expose positively charged peptides (23.4 ± 1.80 mV).

The morphologies of the biomimetic nanovesicles were characterized by transmission electron microscopy (TEM). The TEM micrographs in Fig. 1e show that CLip and CLip-PC@CO-LC NPs display a typical phospholipid bilayered spherical structure, which corroborates previous reports [32, 33, 42]. In addition, we investigated the pH-sensitive characteristics of CLip-PC@CO-LC NPs to verify their pH-triggered cargo release capacity. After incubation at pH 5.0 for 1 h, TEM showed that the structural integrity of CLip-PC@CO-LC NPs began to deteriorate (Fig. 1e).

### Protein markers characterization

The protein profiles of CLip-PC@CO-LC NPs were analyzed using sodium dodecyl sulfate-polyacrylamide gel electrophoresis (SDS-PAGE). The protein profiles of Cm were well-preserved in the prepared CLip and CLip-PC@CO-LC NPs. Additionally, western blotting was performed to analyze the preserved proteins in the CLip-PC@CO-LC NPs. As shown in Fig. 1f, the main cellular membrane marker, Pan-cadherin, was enriched on the surface, whereas the intracellular mitochondrial marker, nuclear marker COXIV, and histone H3 were absent, demonstrating that this purification method can ensure the selective retention of the membrane on the surface of CLip-PC@CO-LC NPs (Fig. 1g).

### Fusogenic property of CLip-PC@CO-LC NPs

Cm and Lipm were stained with DiD and DiO, respectively. The CLSM images show that the C-PC@CO-LC NPs and Lip-PC@CO-LC NPs disperse separately when mixed physically (Fig. 1h). When CLip-PC@CO-LC NPs were prepared using the hybrid membrane CLip, the red and green fluorescence completely merged to display a yellow fluorescence, suggesting the integration of the liposomal membrane and Cm.

### Agarose gel electrophoresis

An N/P ratio of 4 was selected for siRNA loading to guarantee tight condensation of siRNA in the nanocarrier [43, 44]. To further evaluate the characteristics of the stimuli-sensitive delivery systems toward the triggered release of the siRNA load, agarose gel retardation was conducted in the presence or absence of the disulfide-reducing agent dithiothreitol (DTT) at three pH levels: normal (7.4) and acidic (6.5 and 5.0). Agarose gel electrophoresis showed no noticeable movement of siRNA for CLip-PC@CO-LC NPs at different pH levels in the absence of DTT (Fig. 1i). Meanwhile, successful condensation of siRNA in CLip-PC@CO-LC NPs was shown at pH 7.4, whereas a complete mobility shift of siPGAM1 was observed at pH 5.0. This may be attributed to the membrane of the nanocomplexes being completely destroyed upon successful charge conversion as the pH decreased. Furthermore, in the presence of DTT, LC had a weaker siRNA binding affinity.

### Evaluation of DTX release from CLip-PC@CO-LC NPs in vitro

In terms of the discrepant concentration gradient of reductive glutathione (GSH) between the extracellular fluid (2–20 μM) and the tumor cytosol (2–10 mM), reducible disulfide-linked nanocarrier systems have been constructed to trigger intracellular drug release [45, 46]. In addition, based on the gradient of pH levels between the normal tissues (pH 7.4) and slightly acidic TMEs (pH = 6.5–7.2 in tumor tissues; pH = 4.5–6.5 in endosomes and lysosomes), pH-triggered charge-reversal delivery strategies have been widely used to ensure that the cargo load is stable under physiological conditions and to realize pH-triggered intracellular endo/lysosomal quick release [47–49]. As shown in Fig. 1j, approximately 18.7% and 44.2% of DTX was released from CLip-PC@CO-LC NPs without DTT in dialysis media at pH 7.4 at 6 and 48 h, respectively. Anabatic drug release was observed with a single trigger; for example, DTX release reached 43.7% in the presence of 50 mM DTT at pH 7.4 and 42.0% without DTT at pH 5 after 6 h. When CLip-PC@CO-LC NPs were incubated with a release medium at pH = 5 containing DTT, DTX release reached 79.0% with a fast release after 6 h. These results may be attributed to reduction-triggered disulfide bond destruction and the charge-reversal core leading to the disruption of the CLip, indicating that the biomimetic nano-delivery system remains stable in the blood circulation system and has less drug leakage. Nevertheless, the burst release of the drug can be triggered by high GSH and low pH in the TME when the NPs reach the tumor site [50, 51].

### Cellular uptake assay and intracellular distribution analyses

**Fig. 2:**
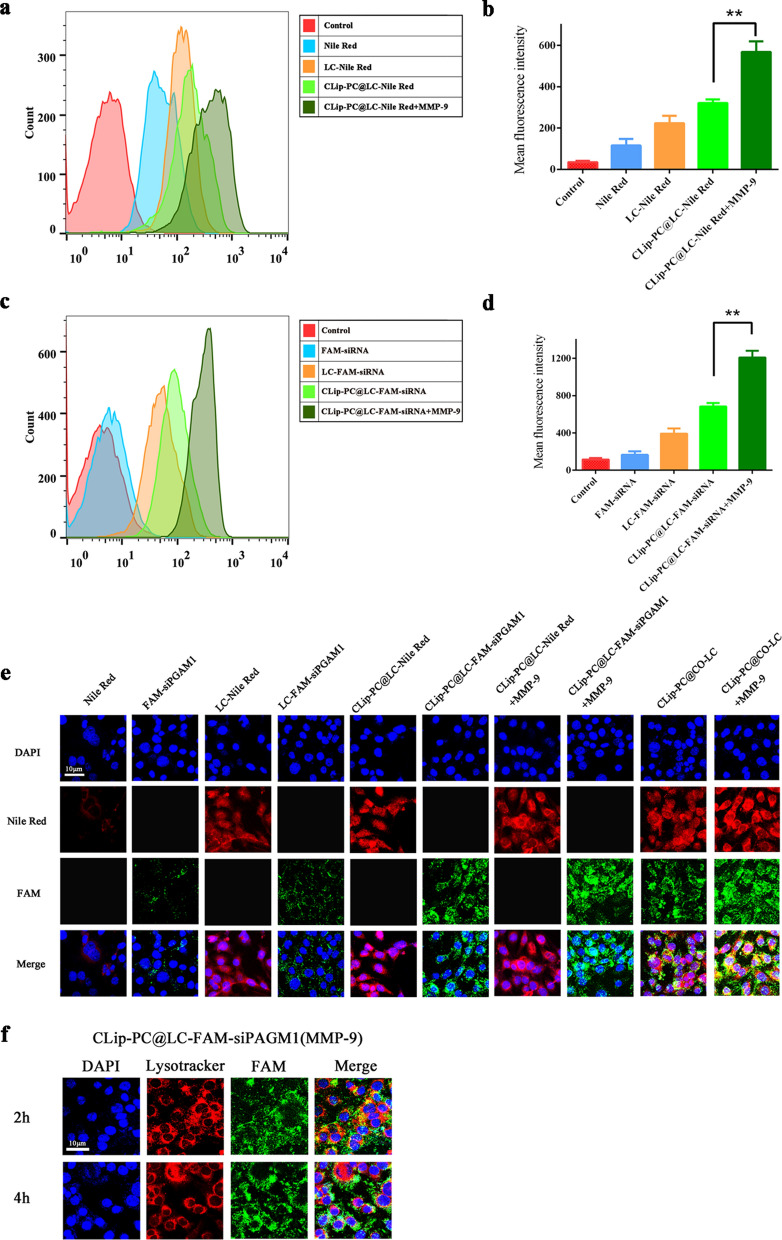
Intracellular NP internalization. **a** Flow cytometry analysis of the amount of Nile red internalized by A549 cells after 4 h treatment. **b** Quantitative analysis of mean fluorescence intensity of Nile red (n = 3). **c** Flow cytometry analysis of the amount of FAM-siPGAM1 internalized by A549 cells after 4 h treatment. **d** Quantitative analysis of mean fluorescence intensity FAM (n = 3). **e** Confocal microscopy images of A549 cells after a 4 h treatment with Nile Red, FAM-siPGAM1, LC-Nile Red, LC-FAM-siPGAM1, CLip-PC@LC-Nile red (with and without 50 nM MMP-9), CLip-PC@LC-FAM-siPGAM1 NPs (with and without 50 nM MMP-9), or CLip-PC@CO-LC NPs (with and without 50 nM MMP-9) for 4 h. Green, FAM-labeled siPGAM1. Red, Nile red. Blue, nuclei dyed with DAPI. Scale bars, 10 μm. **f** Confocal images of A549 cells treated with CLip-PC@CO-LC NPs (MMP-9) for different durations (2 h, 4 h). The green fluorescence of FAM-siPGAM1 is displayed. DAPI (blue) was used to visualize nuclei, and LysoTracker red (red) was used to label the endo/lysosome. Scale bars, 10 μm

To determine whether the MMP-9-switchable peptide-based charge conversion feature of the nanoplatform could enhance cellular internalization of NPs, we substituted Nile red for DTX as a fluorescence probe, and fluorescein amidite (FAM)-labeled siPGAM1 was used to track the siRNA loaded in the NPs. Flow cytometry was used to compare the cellular uptake behavior of CLip-PC@LC-Nile red NPs (pretreated with or without MMP-9), free Nile red, and LC-Nile red after a 4-h incubation. The results showed that CLip-PC@LC-Nile red NPs (pretreated with MMP-9) displayed higher uptake levels than did CLip-PC@LC-Nile red NPs without additional MMP-9 (p < 0.01). Tumor cell membranes endowed our nanoplatform with tumor-targeting via homologous binding. As expected, LC-Nile red NPs were internalized in A549 cells more efficiently than free Nile red. Furthermore, the cellular uptake behavior of siPGAM1 mediated by the biomimetic nanoplatform was also evaluated. As shown in Fig. 2c, d, flow cytometry analysis revealed that almost no FAM-positive cells were observed for siPGAM1 in its native form. However, the uptake rate of CLip-PC@LC-FAM-siPGAM1 NPs (without MMP-9) was higher compared to that of free siPGAM1 (p < 0.001). In addition, the uptake rate of CLip-PC@LC-FAM-siPGAM1 NPs (pretreated with MMP-9) was higher than that of CLip-PC@LC-FAM-siPGAM1 NPs without MMP-9 treatment (p < 0.01).

**Fig. 3:**
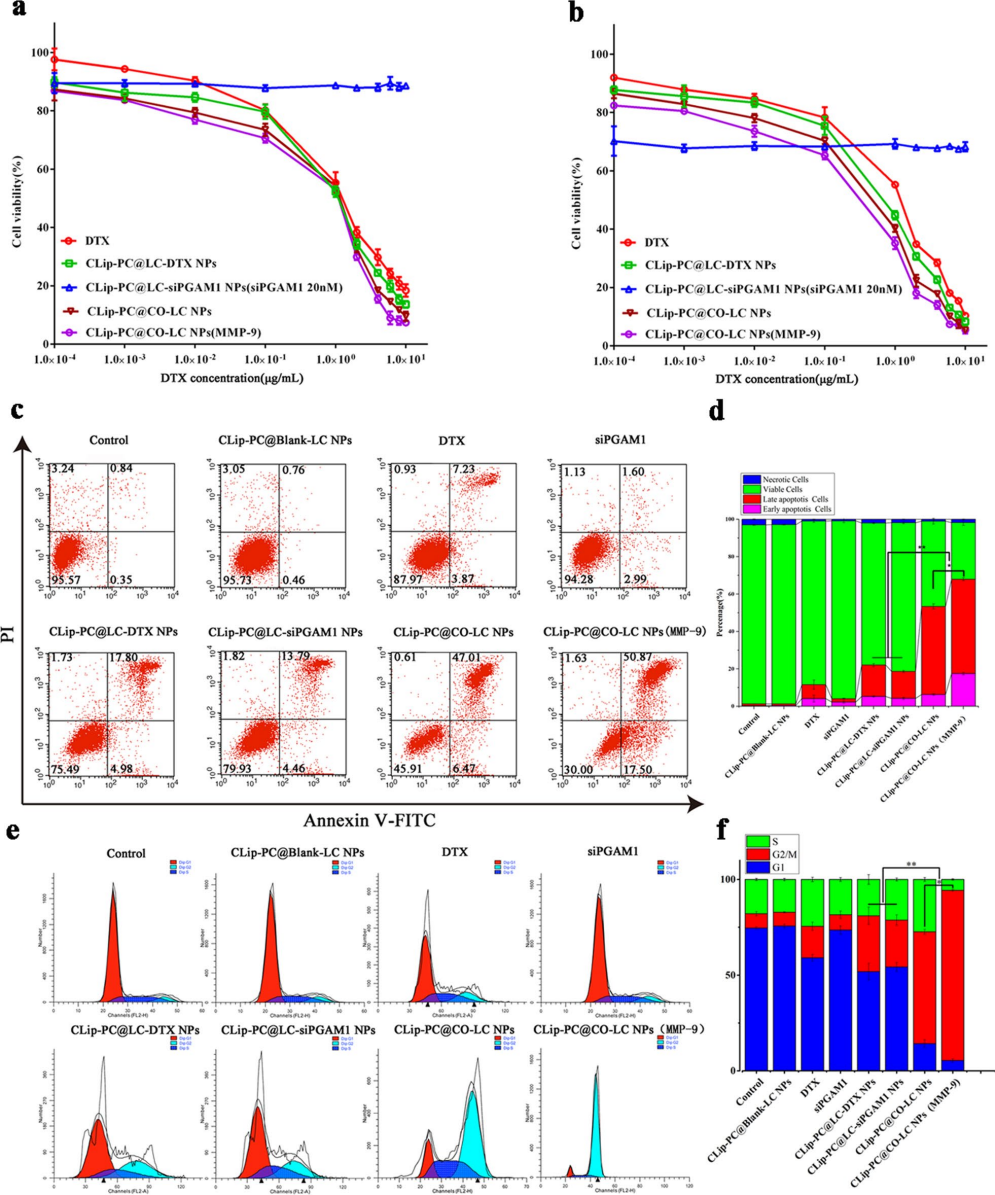
In vitro therapeutic efficacy of CLip-PC@CO-LC NPs. Dosage-dependent response of cell viability in A549 cells after treatment with DTX, CLip-PC@LC-DTX NPs, CLip-PC@LC-siPGAM1 NPs, CLip-PC@CO-LC NPs, or CLip-PC@CO-LC NPs (MMP-9) for **a** 24 h and **b** 48 h; data are represented as mean ± standard deviation (SD; n = 5). **c** Fluorescence-activated cell sorter analysis of apoptosis after annexin V-fluoresceine isothiocyanate/propidium iodide (FITC/PI) staining. **d** Percentages of apoptotic and necrotic cells were determined using Annexin V-PI; mean ± SD (n = 3). **e** Histograms showing the percentage of A549 cells in different phases of the cell cycle after a 48 h treatment with different DTX formulations. **f** S, G2/M, G1 phase distributions of various groups (results are shown as mean ± SD, n = 3)

CLSM was performed to evaluate the intracellular distribution of Nile red and FAM-siPGAM1. The red and green fluorescence imaged by CLSM represented the fluorescence of Dox and FAM-siPGAM1, respectively, indicating the effective delivery of CLip-PC@CO-LC NPs into A549 cells within 4 h (Fig. 3e). The nuclei of A549 cells were stained with 4′,6-diamidino-2-phenylindole (DAPI) (blue fluorescence). The merged yellow color imaged by CLSM represents the overlap of green fluorescence and red fluorescence from Dox with FAM-siPGAM1. Stronger red fluorescence was displayed in CLip-PC@LC-Nile red and CLip-PC@LC-Nile red (MMP-9) compared with that in free Nile red group (Fig. 3e). Compared with the CLip-PC@LC-FAM-siPGAM1 NP group, the free FAM-siPGAM1 NP group exhibited negligible fluorescence, which supported the findings obtained by flow cytometry (Fig. 3b, c). Moreover, after internalization for 4 h, the CLip-PC@CO-LC NP group showed that the green fluorescence of FAM overlapped with the red fluorescence of Nile red and was mainly distributed near the nucleus. Additionally, the intracellular drug delivery efficiency of Dox and siPGAM1 was investigated using CLip-PC@CO-LC NPs with or without MMP-9 pre-treatment. Compare with that in the CLip-PC@CO-LC NP group, stronger red and green fluorescence emerged in CLip-PC@CO-LC NP (MMP-9)-treated A549 cells (Fig. 2e). These results may be attributed to the cleavage of the MMP-9-sensitive peptide from the surface of CLip by MMP-9 that exposed the positively charged CPP, thus significantly enhancing its penetration and uptake [52, 53].

### Intracellular lysosome escape assay

Effective endosomal escape is the key step in achieving effective intracellular delivery of siRNA by nonviral nanocarriers [54]. Therefore, escape from late endosomes/lysosomes is a crucial step for siRNA to perform gene knockdown; here, a pH-sensitive charge-reversible method was designed to achieve charge reversion, induce the rupture of endosomal membranes at endosomal pH values, and effect siRNA delivery into the cytoplasm and gene knockdown [40, 55]. In this study, LysoTracker red dye was used to label intracellular acidic lysosomes. As shown in Fig. 2f, the merged yellow color represents the overlapped signal of Lysotracker red dye (red fluorescence) with FAM-siPGAM1 (green fluorescence), indicating that FAM-siPGAM1 was trapped in the endosome. Notably, most CLip-PC@LC-FAM-siPAGM1 (MMP-9) NPs remain trapped inside the lysosome after 2 h, while the green fluorescence was separated from the red region after 4 h (Fig. 2f), indicating that CLip-PC@LC-FAM-siPAGM1 (MMP-9) exhibits endo/lysosome escape capability with the help of the charge-reversal core. These results are in agreement with those of past reports of PC successfully realizing lysosome escape of siRNA because of its pH-triggered charge conversion [39].

### In vitro cytotoxicity and biocompatibility evaluation

A cell viability assay was conducted to investigate the cytotoxicity of A549 cells, while cell proliferative capability was assessed using CCK-8 assay. As shown in Additional file 1: Figure S3, no significant cell toxicity was observed in A549 cells after the 24 or 48 h treatment with CLip-PC@BLank-LC NPs (0–150 μg/mL), which indicated the low cytotoxicity and adequate biocompatibility of these biomimetic NPs. Among groups incubated with DTX, CLip-PC@LC-DTX NPs, CLip-PC@CO-LC NPs, and CLip-PC@CO-LC NPs (MMP-9) inhibited the proliferation of A549 cells in a dose- and time-dependent manner. As illustrated in Fig. 3a, b, compared with free DTX, CLip-PC@LC-DTX NPs exhibited a stronger inhibitory effect at almost all concentrations, likely due to the CLip-mediated homogenous targeting and the continuous release of DTX from CLip-PC@LC-DTX NPs. The IC_50_ in the CLip-PC@CO-LC NP (MMP-9) group was lower than that in the CLip-PC@CO-LC NP group (0.33 μg/mL vs. 0.45 μg/mL at 24 h; 0.12 μg/mL vs. 0.23 μg/mL at 48 h), indicating that the MMP-9-activated biomimetic nanosystem CLip-PC@CO-LC NPs could boost the antiproliferation effect of CLip-PC@CO-LC NPs in the presence of MMP-9. The IC_50_ in A549 cells was the lowest in the CLip-PC@CO-LC NP (MMP-9) group, approximately 3.10- and 6.20-fold lower than that in the free DTX group after 24 and 48 h, respectively, indicating that co-delivery of DTX and siPGAM1 led to inhibition of cell proliferation. In addition, the IC_50_ in the CLip-PC@CO-LC NPs group was lower than that in the CLip-PC@LC-DTX NPs group (0.33 μg/mL vs. 0.70 μg/mL at 24 h; 0.12 μg/mL vs. 0.45 μg/mL at 48 h), suggesting synergistic antiproliferative effects of DTX and siPGAM1 against A549 cell in vitro.

### Cell apoptosis and cycle assays

To further evaluate apoptosis in A549 cells, annexin V-fluoresceine isothiocyanate/propidium iodide (FITC/PI) was used for the double staining of cells. As indicated in Fig. 3a, compared with the control, A549 cells exposed to CLip-PC@Blank-LC NPs showed no obvious apoptosis after 48 h of treatment, which is in agreement with the results of the cell viability analysis. CLip-PC@LC-DTX NPs caused cell apoptosis in 22.78% of the cells, a rate approximately twofold higher than that in the free DTX group. The total apoptosis rate of CLip-PC@CO-LC NPs was more effective than that of CLip-PC@LC-DTX NPs by approximately 2.35-fold. Naked siPGAM1 induced cell apoptosis at a rate of 4.59%, whereas CLip-PC@LC-siPGAM1 NPs had a cell apoptosis rate of approximately 18% in A549 cells, suggesting that siPGAM1 could more effectively induce cell apoptosis when loaded with our biomimetic nanocarrier. Moreover, CLip-PC@CO-LC NPs (MMP-9) were more effective in cell apoptosis than CLip-PC@CO-LC NPs (apoptosis rate: 68.37% vs. 53.48%). This might be because the CLip-PC@CO-LC NPs could promote its uptake and intracellular drug release. These results illustrate that co-loading with our TME-activated biomimetic nanosystem could knock down PGAM1, inhibit glycolysis when used in combination with DTX chemotherapy, and cause significantly more apoptosis than mono-chemotherapy, which is in accord with the findings of previous studies [21, 56].

The main mechanism underlaying the anticancer effect of DTX is the prevention of microtubule depolymerization, which causes G2/M phase cell cycle arrest [57]. As shown in Fig. 3c, the CLip-PC@Blank-LC NPs showed no increase in the proportion of A549 cells in the G2/M phase, which is consistent with the suitable biocompatibility of the CLip-PC@Blank-LC NPs, as shown by the cell viability assay. Compared with the blank control, naked siPGAM1 could not cause G2/M phase cell cycle arrest (7.51% ± 1.07% vs. 7.97 ± 1.99%), while the CLip-PC@LC-siGAM1 NPs caused partial accumulation in the G2/M phase of the cell cycle (3.27-fold vs. control), indicating that naked siPGAM1 lacked therapeutic function in vitro unless delivered by the biomimetic nanocarrier. This is consistent with a study showing that PGAM1 silencing could delay cell cycle progression caused by unrepaired DNA lesions [58]. As expected, free DTX caused partial accumulation of cells in the G2/M phase after 48 h of incubation (2.18-fold vs. control), while the CLip-PC@LC-DTX NPs caused substantial accumulation after 48 h of incubation (3.89-fold vs. control). In the CLip-PC@CO-LC NP and CLip-PC@CO-LC NP (MMP-9) groups, the proportions of cells in the G2/M phase were 58.45 ± 1.27 and 88.9% ± 0.51, respectively (p < 0.01). These results indicate that co-delivery of DTX and siPGAM1 in our biomimetic nanocarrier synergistically promoted cell cycle arrest.

### Measurement of intracellular lactate, ATP level, and glucose uptake

**Fig. 4:**
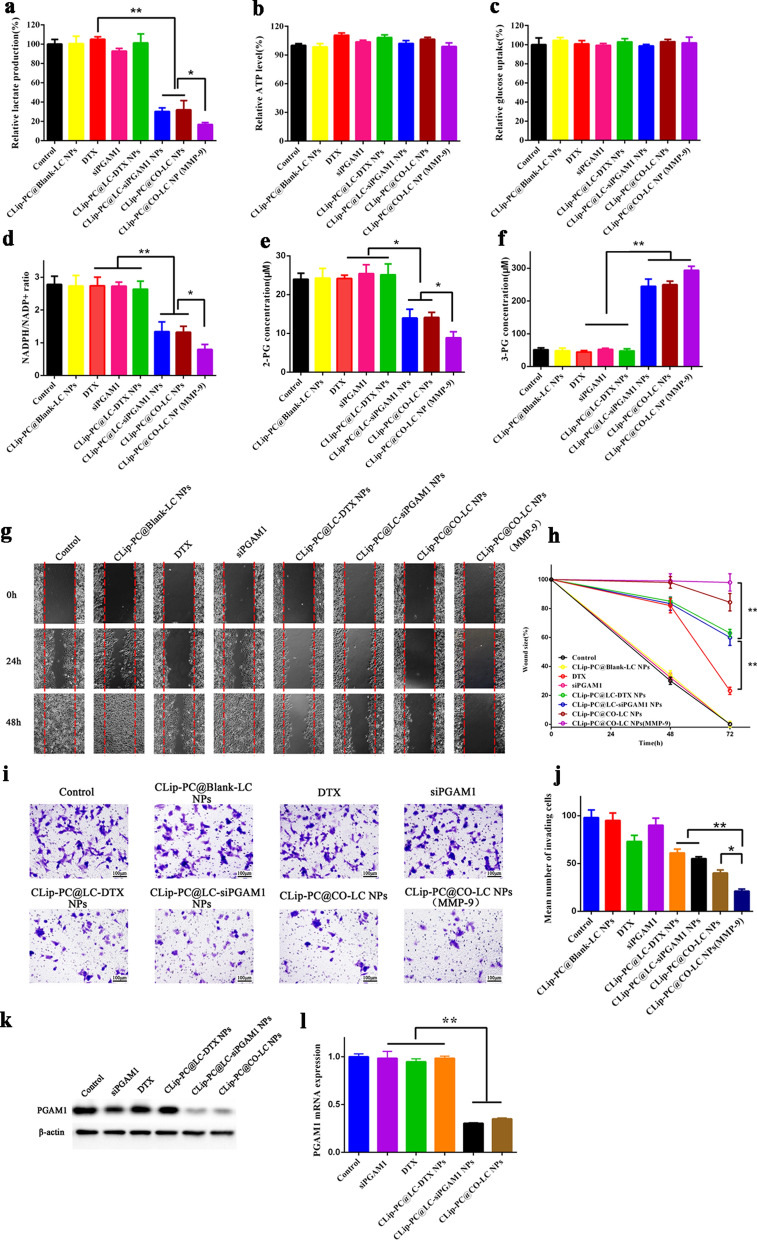
Inhibitory effects of CLip-PC@CO-LC NPs on glycolysis, cell migration, and invasion abilities in A549 cells. **a** Intracellular lactate levels and **b** ATP levels of A549 cells after 6 h treatment with CLip-PC@Blank-LC NPs, DTX, siPGAM1, CLip-PC@LC-DTX NPs, CLip-PC@LC-siPGAM1 NPs, CLip-PC@CO-LC NPs, or CLip-PC@CO-LC NP (MMP-9). The data are shown as mean ± standard deviation (SD; n = 3). **c** Normalized glucose uptake of A549 cells after 6 h of incubation with different formulations. The data are shown as mean ± SD (n = 3). **d** Intracellular NADPH/NADP^+^, **e** 2-PG, and **f** 3-PG were measured after 24 h of incubation with different formulations. The data are shown as mean ± SD (n = 3). **g** Wound healing analysis of A549 cells treated with CLip-PC@Blank-LC NPs, DTX, siPGAM1, CLip-PC@LC-DTX NPs, CLip-PC@LC-siPGAM1 NPs, CLip-PC@CO-LC NPs, or CLip-PC@CO-LC NPs (MMP-9) for 0, 24, and 48 h; scale bar = 100 μm (mean ± SD, n = 3). **h** Quantitative analysis of the migration rate in A549 cells for 48 h. **i** Typical images of Transwell assays of CLip-PC@Blank-LC NPs, DTX, siPGAM1, CLip-PC@LC-DTX NPs, CLip-PC@LC-siPGAM1 NPs, CLip-PC@CO-LC NPs, and CLip-PC@CO-LC NPs (MMP-9) in A549 cells (scale bar = 100 μm). **j** Quantitative analysis of invading cells in each group. The quantitative data are presented as mean ± SD (n = 3). *P < 0.05, **P < 0.01, ***P < 0.001. **k** Western blotting assays to determine PGAM1 expression in A549 cells with various treatments. **l** RT-PCR quantification of *PGAM1* mRNA expressed in A549 cells in each group

Lactate is an important final metabolite of glycolysis [59]. After the *PGAM1* gene was effectively silenced, the glycolysis pathway was inhibited, which reduced the consequent production of lactate. As illustrated in Fig. 4a, the in vitro experiments showed that lactate production was significantly lower in the CLip-PC@LC-siPGAM1 NP, CLip-PC@CO-LC NP, and CLip-PC@CO-LC NP (MMP-9) groups than in the DTX-treated A549 cells (p < 0.05). The ATP level and glucose uptake rate of A549 cells after different treatments were quantitatively measured. As shown in Fig. 4b, c, inhibition of PGAM1 hardly affected the glucose uptake rate or intracellular ATP levels. This might be due to the attenuation of PGAM1 inhibition of glycolysis, PPP, and biosynthesis, which was simultaneously compensated for by alternative mechanisms other than mitochondrial oxidative phosphorylation simultaneously [15].

### NADPH/NADP+ ratio and intracellular 2- and 3-PG concentration measurement

PGAM1 inhibition could result in a decreased NADPH/NADP+ ratio and oxidative PPP flux [60, 61]. Hence, we investigated the influence of CLip-PC@CO-LC NPs on the NADPH/NADP^+^ ratio. The ratios were remarkably lower in the CLip-PC@LC-siPGAM1 NP, CLip-PC@CO-LC NP, and CLip-PC@CO-LC NP (MMP-9) groups compared with those in the groups treated with DTX, siPGAM1, or CLip-PC@LC-DTX NPs (p < 0.05; Fig. 4d).

Moreover, PGAM1, one of the most important enzymes in cancer metabolism, catalyzes the conversion of 3-PG to 2-PG during aerobic glycolysis [15, 62]. We next examined the effect of CLip-PC@CO-LC NP-altered 2- and 3-PG levels on cancer cell metabolism. As illustrated in Fig. 4e, f, attenuation of PGAM1 by CLip-PC@LC-siPGAM1, CLip-PC@CO-LC NPs, and CLip-PC@CO-LC NPs (MMP-9) in A549 cells not only led to decreased 2-PG (Fig. 4e) but also increased 3-PG (Fig. 4f) levels compared with those in corresponding control cells treated with DTX, siPGAM1, or CLip-PC@LC-DTX NPs. These results further indicated that effectively silencing the PGAM1 gene by CLip-PC@LC-siPGAM1 or CLip-PC@CO-LC NPs could control the metabolite levels of its substrate 3-PG and product 2-PG in cancer cells. This is attributed to PGAM1-dependent coordination of glycolysis and anabolic biosynthesis.

### Transwell migration and invasion assays

To explore the effect of CLip-PC@CO-LC NPs on NSCLC metastasis, wound healing and Transwell invasion assays were used to examine the impact of CLip-PC@CO-LC NPs on cell migration and invasion, respectively. As illustrated in Fig. 4g, h, after incubation for 48 h, the wound size of A549 cells treated with DTX, CLip-PC@LC-DTX NPs, CLip-PC@LC-siPGAM1 NPs, or CLip-PC@CO-LC NPs were 23.20% ± 2.50, 63.0% ± 2.6, 60.0% ± 5.5, and 84.3% ± 6.0, respectively. The wounds of A549 cells treated with free siPGAM1 failed to close after 48 h. CLip-PC@CO-LC NPs (MMP-9) showed stronger migration suppression compared with that of CLip-PC@CO-LC NPs in A549 cells. These results suggest that, after co-delivery of DTX with siPGAM1 by the CLip-PC@CO-LC NPs, the migration behavior of A549 cells can be effectively suppressed. Then, a Transwell assay was performed to further observe the impact of CLip-PC@CO-LC NPs on the invasion and metastasis abilities of A549 cells. As illustrated in Fig. 4i, j, CLip-PC@Blank-LC NPs and siPGAM1 showed slight anti-cell migration activity compared with that in the cells treated with a normal medium. However, in the DTX, CLip-PC@LC-DTX NP, CLip-PC@LC-siPGAM1 NP, and CLip-PC@CO-LC NP groups, the number of cells migrating across the Transwell membrane decreased to 74.5%, 62.2%, 56.1%, and 40.8%, respectively, from the number that migrated with the negative control; thus, the number was drastically reduced by 78.5% in the CLip-PC@CO-LC NPs (MMP-9) group, indicating that co-delivery of DTX and siPGAM1 by our TME-activated biomimetic nanosystem could reduce the migratory and invasive abilities of A549 cells. These results illustrate the great potential of CLip-PC@CO-LC NPs (MMP-9) in inhibiting the metastatic behavior of A549 cells in vitro.

### In vitro siPGAM1 transfection

The protein expression levels of PGAM1 were detected in the six groups using Western blotting. As illustrated in Fig. 4k, the expression levels of PGAM1 were significantly reduced in the CLip-PC@LC-siPGAM1 and CLip-PC@CO-LC NP groups compared with those in the control, siPGAM1, DTX, and CLip-PC@LC-DTX NP groups. These findings indicate that the translation of PGAM1 mRNA was downregulated in A549 cells by siPGAM1 delivery using our biomimetic nanocarrier. Furthermore, to investigate the regulatory effect of siPGAM1 on PGAM1 expression, the level of PGAM1 mRNA was determined using quantitative reverse transcription-PCR (RT-PCR). The results showed that after treatment with CLip-PC@LC-siPGAM1 NPs or CLip-PC@CO-LC NPs, *PGAM1* mRNA levels substantially declined compared with those in the DTX and CLip-PC@LC-DTX NP groups (p < 0.05) (Fig. 4l). Taken together, these results suggest that the co-loading of siPGAM1 with DTX by our biomimetic nanocarrier effectively inhibits *PGAM1* expression.

### Biodistribution

**Fig. 5:**
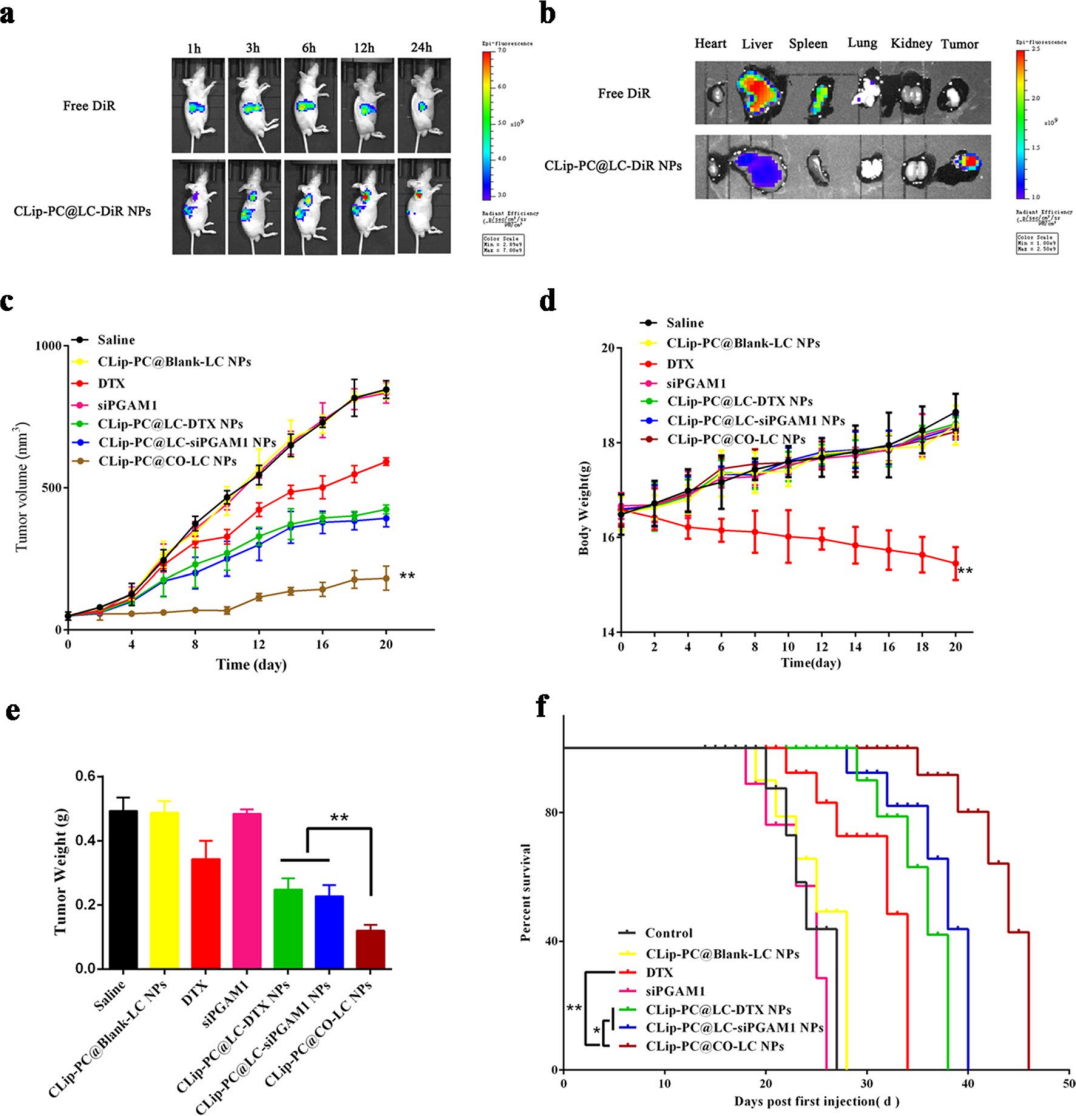
Biodistribution and antitumor activity of CLip-PC@CO-LC NPs. **a** Biodistribution of free DiR and CLip-PC@LC-DiR in A549 cell tumor-bearing nude BALB/c mice at 1, 3, 6, 12, and 24 h after administration. **b** Fluorescence distribution images of tumors and major organs after intravenously injecting free DiR and CLip-PC@LC-DiR NPs in mice tails. **c** Tumor growth curves of A549 tumor-bearing mice after receiving various treatments (n = 6). **d** Body weights of the A549 tumor-bearing mice after receiving various treatments. **e** The tumor weights of the A549 tumors excised from all groups after different treatments. Data are expressed as the mean ± standard deviation (SD; n = 6). **f** Mice survival rates after different treatments (n = 6)

DiR, a lipophilic dye with strong absorption in the near-infrared region, has been extensively used as a tracer in the bioimaging of NP distribution [63, 64]. As shown in Fig. 5a, live mice were monitored at 1, 3, 6, 12, and 24 h post-injection by in vivo fluorescence imaging. Biodistribution images indicated that the CLip-PC@LC-DiR NPs accumulated in the A549 tumor xenografts at 3 h after administration. Strong fluorescence signals were detected even at 24 h post-injection, showing the gradual decay of the fluorescence signal in the liver, which demonstrated an accumulation enhancement by targeting. However, decreased fluorescence was displayed at the tumor site; fluorescence was detected only in the liver in the free DiR group. Ex vivo fluorescence distribution in tumors and other major organ tissues was also observed (Fig. 5b), demonstrating that the liver and spleen were the main organs in which fluorescence accumulated in the free DiR group. The fluorescence signal of CLip-PC@LC-DiR NPs was mainly distributed at the tumor site. These findings demonstrate that CLip-PC@LC-DiR NPs possess excellent targeting features in vitro and in vivo owing to the long blood retention time, homologous targeting ability, and characteristics of the TME-activated by NPs with multifunctional integrated hybrid nanovesicle coating.

### Antitumor effect in vivo

The synergistic in vivo inhibition of tumor growth by co-delivery using CLip-PC@CO-LC NPs was evaluated in a xenografted nude mouse model. The tumor volumes and body weights of the mice were monitored and recorded at regular intervals (Fig. 5c, d). We found that the treatment groups other than the CLip-PC@Blank-LC and free siPGAM1 groups exhibited higher antitumor efficacy than that in the saline group (p < 0.01) and that DTX exhibited poor inhibitory effects. The volumes of the extracted tumors and the tumor weight histograms showed that CLip-PC@LC-DTX NPs and CLip-PC@LC-siPGAM1 NPs exhibited enhanced tumor inhibition efficiency compared with that in the DTX group due to their tumor targeting and TME responsive capabilities (Fig. 5c, e). At the end of the observation period, the size of the tumors in the CLip-PC@CO-LC NP group was 2.26-, 1.16-, and 1.33-fold smaller than those in the free DTX, CLip-PC@LC-siPGAM1 NP, and CLip-PC@LC-DTX NP groups, respectively, which indicated the in vivo tumor cooperative eradicating capability of DTX in combination with siPGAM1. In particular, the overall inhibitory rates by tumor volume were 31.9%, 56.8%, 53.1%, and 83.5% for the DTX, CLip-PC@LC-siPGAM1 NP, CLip-PC@LC-DTX NP, and CLip-PC@CO-LC NP groups, respectively, which illustrates the effectiveness of multifunctional integrated hybrid nanovesicle-mediated co-delivery. These findings support the results of in vitro cell assays.

Except for that in the free DTX group, the body weight of mice showed no noticeable change during the treatment period (Fig. 5d), demonstrating no noticeable systemic toxicity from our biomimetic nanocarrier. Additionally, we examined the survival of the treated mice and found, as shown in Fig. 5e, that the median survival of the CLip-PC@CO-LC NP group reached 46 days vs. 27 days in the saline group, 28 days in the CLip-PC@BLank-LC NP group, and 34 days in the DTX group, suggesting that CLip-PC@CO-LC NPs potently suppressed tumor growth and significantly prolonged animal survival.

### Histology and immunohistochemistry (IHC) analysis

**Fig. 6:**
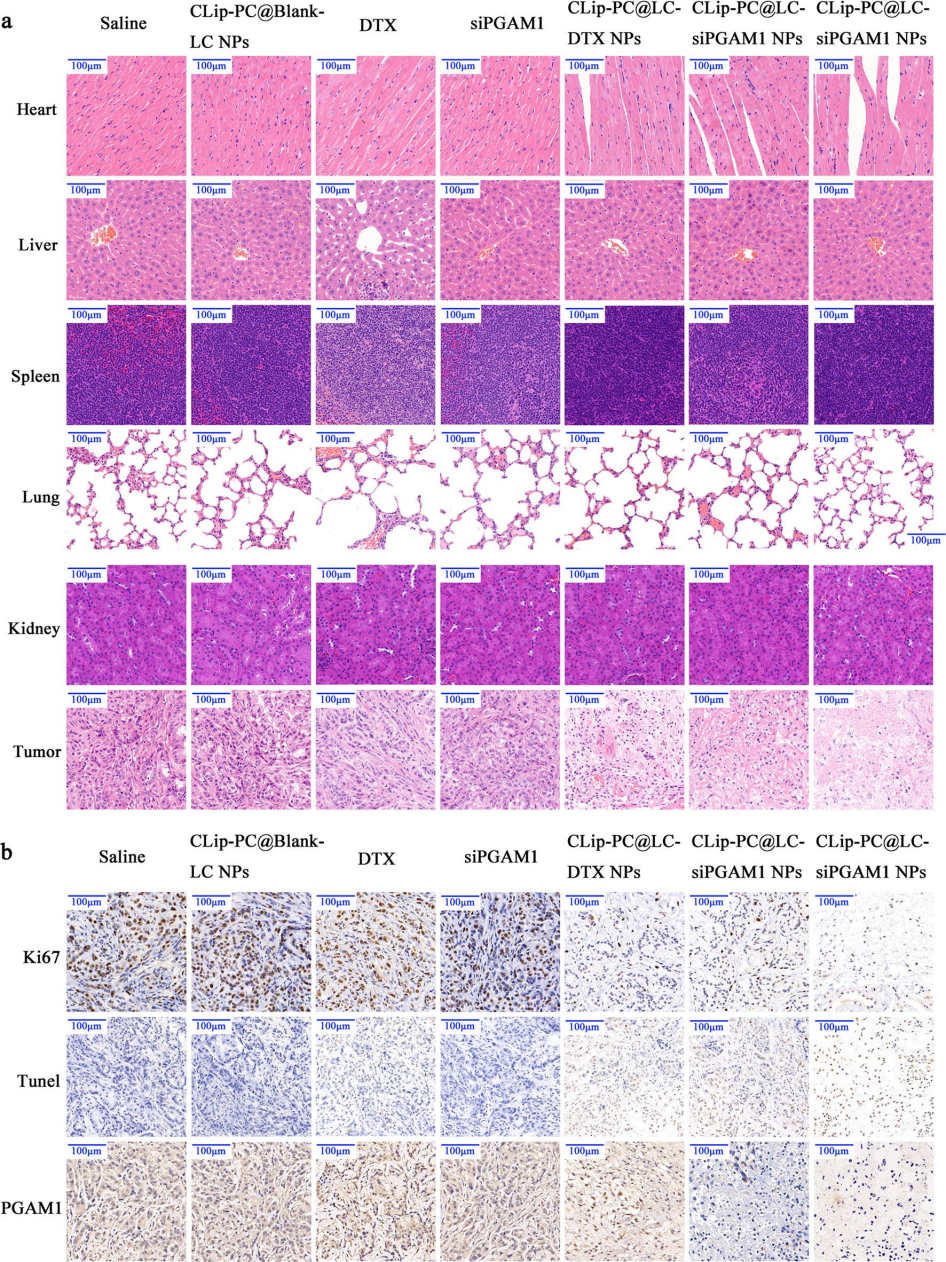
In vivo safety and tumor-suppressive effects. **a** hematoxylin–eosin-stained slices of major organs and tumors collected from each group. **b** Immunohistochemistry analysis of Ki67, TUNEL, and PGAM1 expression in tumor sections. Scale bar, 100 μm

The biosafety of CLip-PC@CO-LC NPs was observed by hematoxylin and eosin (HE) staining in vivo. As shown in Fig. 6 a, the histological analyses indicated that CLip-PC@Blank-LC NPs, CLip-PC@LC-DTX NPs, CLip-PC@LC-siPGAM1 NPs, and CLip-PC@CO-LC NPs exhibited no overt histological changes or functional damage to normal organs, whereas significant pathological damage to liver tissue was observed in the free DTX group. In addition, CLip-PC@CO-LC NPs exhibited markedly more dramatic tumor necrosis than both the CLip-PC@LC-DTX NP and CLip-PC@LC-siPGAM1 NP groups, which demonstrated that DTX and siPGAM1 exhibited synergistically high inhibition efficiency against the growth of A549 xenografts in vivo.

Ki67, a proliferation marker, was detected to further evaluate the anti-cancer efficacy in each group [65]. As shown in Fig. 6a, treatment with CLip-PC@CO-LC NPs showed the lowest Ki67 expression in A549 cells, indicating that it was the most effective in reducing tumor cell proliferation, which supported the results of the in vivo studies. Tumor section apoptosis was evaluated via TdT-mediated dUTP nick-end labeling (TUNEL) staining after treatment (Fig. 6b). Free DTX- and CLip-PC@LC-DTX NP-treated cells showed a comparable number of apoptotic cells, which was remarkably higher than that in the saline group. Moreover, compared with that of the CLip-PC@LC-DTX NPs and CLip-PC@LC-siPGAM1 NPs, the co-delivery system of the CLip-PC@CO-LC NPs significantly increased cell apoptosis and displayed the most significant effect. As shown in Fig. 6b, siPGAM1 loading by CLip-PC@LC-siPGAM1 NPs significantly reduced PGAM1 expression in tumors, with decreased tumor cell proliferation and increased apoptosis, which were observed in the CLip-PC@CO-LC NP group. These results suggest that the CLip-PC@CO-LC NPs showed significant suppression of tumor growth attributed to the co-loading of DTX and siPGAM1 via CLip-PC@CO-LC NPs, which very efficiently targeted A549 lung tumors, displayed extraordinary antitumor activity, and reduced systemic toxicity and undesired off-target toxicity.

## Conclusions

In this study, we successfully fused A549 Cm with MMP-9-switchable peptide-based charge-reversal liposome membranes and prepared integrated hybrid nanovesicle–cancer liposome-coated NPs for treating lung cancer in vivo. The integrated hybrid nanovesicle possesses unique and desirable features, including extended circulation half-life, effective homogeneous lung cancer-targeting ability, excellent biocompatibility, high tumor accumulation, MMP-9-activated tumor cell penetration, pH-triggered membrane disruption, and redox-triggered DTX and siRNA release. This cancer liposome-based nanovesicle, developed for co-loading of siPGAM1 and DTX, exhibits a synergistic tumor inhibition effect both in vitro and in vivo by regulating glycolysis, showing no notable toxicity and successfully prolonging the lifespan of xenografted mice. The proposed strategy may be adapted to integrate artificially functionalized lipid membranes with various natural cell membranes and enable the release of multiple TME-sensitive drugs for the treatment of different cancers.

## Methods

### Materials

DTX (98% purity) and lipoic acid (99% purity) were obtained from BBI Life Science Corporation (Shanghai, China). LC was synthesized using the F-moc-solid-phase peptide synthesis method, as previously described [29]. PGAM1 monoclonal antibody (67470-1-Ig) was purchased from Proteintech Group (Rosemont, IL, USA). 1,2-dipalmitoyl-sn-glycero-3-phosphocholine (DPPC, 99% purity) was purchased from Xi'an Ruixi Biological Technology Co., Ltd (Xi’an, China). Cholesterol (99% purity) was purchased from Sigma-Aldrich (St. Louis, MO, USA). DSPE-PEG-MAP (DSPE-PEG2000-RRRRRRRRR-PVGLIG-EGGEGGEGG) and poly-l-lysine (98% purity) were purchased from Chinese Peptide Co., Ltd. (Zhejiang, China). CA (citraconic anhydride) (98% purity) was obtained from Aladdin (Shanghai, China). A549 cells were purchased from the Institute of Biochemistry and Cell Biology (Shanghai, China). Negative siRNA (siNeg) control (sense, 5ʹ-UUCUCCGAACGUGUCACGUdTdT-3ʹ; antisense, 5ʹ-ACGUGACACGUUCGGAGAAdTdT-3ʹ) and siPGAM1 (sense, 5ʹ-CGACUGGUAUUCCCAUUGUTT-3ʹ; antisense, 5ʹ-ACAAUGGGAAUACCAGUCGTT-3ʹ) were synthesized by GenePharma (Shanghai, China). FAM dyes were introduced to the 5ʹ-end of the antisense strand.

### Animals

All animal experiments were conducted according to the United States National Institutes of Health Guide for the Care and Use of Laboratory Animals (NIH Publication No. 8023, revised 1978), and the animal experimental procedures were approved by the Animal Ethics Committee of Shanghai Pulmonary Hospital of Tongji University.

### Preparation of CLip

A549 cell membrane material was prepared as previously described [41, 66]. Briefly, human A549 cells were harvested, washed with PBS, and centrifuged. Then, phenylmethanesulfonyl fluoride (PMSF) was added to a final concentration of 1 mM before the use of membrane protein extraction reagent A to resuspend the collected A549 cells, which were then placed in an ice bath for 15 min. The cells were freeze-thawed three times. The precipitate was then removed by centrifugation at 700×*g* for 10 min at 4 °C, and the membrane was separated from the supernatant by centrifugation at 14,000×*g* for 30 min at 4 °C. Finally, A549 Cm were stored at 4 °C. The thin-film hydration method was used to fabricate the CLips, followed by extrusion, as formerly reported [32, 67]. Briefly, 1.3 mg of DPPC, 0.3 mg of cholesterol, and 2.4 mg of DSPE-PEG-MAP were dissolved in dichloromethane (5 mL), and the resulting mixture was evaporated in a round-bottom flask under vacuum at 50 °C in a rotary evaporator (Rotavapor R-100, Buchi, Switzerland) to obtain a thin film. The lipid film (4 mg) was hydrated with 1.85 mL of 10% sucrose and 150 μL of A549 Cm suspension or with 2 mL of 10% sucrose solution to prepare CLip or Lipm. Further, the suspension was sonicated in an ice bath for 3 min and then extruded through 0.2 and 0.1 μm polycarbonate membranes (Whatman, Maidstone, UK) at room temperature (RT) using an extruder system (Avanti Polar Lipids, Alabaster, AL, USA). The CLips were stored at 4 °C until use.

### Membrane fusion analysis

To verify whether Lipm was successfully incorporated into Cm, FRET assay was conducted to verify membrane integration as previously described [41]. Briefly, Cm was labeled with DOPE-RhB (excitation/emission = 560/583 nm) and C6-NBD (excitation/emission = 460/534 nm). Lipm was then added to the dye-labeled Cm at different weight ratios (5:1, 4:1, 3:1, 2:1, 1:1, and 0:1) for hydration, sonicated in an ice bath for 3 min, and then extruded through 0.2 and 0.1 μm polycarbonate membranes. The fluorescence spectrum of the dye-labeled CLip was recorded in the range of 500 to 650 nm at an excitation wavelength of 470 nm. The fluorescence recovery of the donor (C6-NBD) was used to observe the membrane fusion. FTIR spectroscopic analysis of the lyophilized CLip samples was conducted using a VERTEX 70 instrument (Bruker, Bremen, Germany).

### Synthesis of PC

Poly-l-lysine (50 mg) was dissolved in 80 mL phosphate-buffered saline (PBS, pH = 7.4); then, CA (87.4 mg, 0.77 mmol) was slowly added, and the pH value of the solution was adjusted to neutral with aqueous NaOH solution (5 M). After stirring overnight at RT, we dialyzed the resulting solutions against distilled water by using a dialysis membrane (MWCO = 500, Spectrum Laboratories, Rancho Dominguez, CA, USA) for 72 h and then performed lyophilization. The structure of the final product was determined by ^1^H NMR spectroscopy in D_2_O at 600 MHz.

### Preparation of CLip-PC@CO-LC NPs nanocomplexes and characterization

DTX-loaded LC NPs (LC/DTX) were prepared as previously reported [29]. CO-LC NPs and siPGAM1-loaded LC NPs (LC/siPGAM1) were fabricated by adding exactly the right proportion of siPGAM1 to LC/DTX NPs and Blank-LC NPs, respectively, which were mixed at an N/P ratio of 4, vortexed for 30 s, and incubated for 30 min at RT before use. Thereafter, PC@CO-LC NPs were fabricated by adding exactly the right proportion of PC (1 mg/mL) to Co-LC NPs, followed by incubation for 30 min at RT; the molar ratio of the carbonyl group in PC and the amino group in LC in this reaction was 3:1. To fuse the hybrid membrane CLip, the mixture of the tumor cell membrane and lipid film at a membrane protein-to-liposome weight ratio of 1:1 was sonicated for 2 min. Finally, to encapsulate the fused hybrid membrane CLip over PC@CO-LC NPs, the mixture was sonicated for 2 min in an ice bath using an FS30D bath sonicator (Thermo Fisher Scientific, Waltham, MA, USA). The zeta potentials and hydrate particle sizes of CO-LC NPs, PC@CO-LC NPs, and CLip-PC@CO-LC NPs were detected by DLS (Zetasizer Nano ZS90, Malvern).

To investigate the sensitivity of CLip-PC@CO-LC NPs to acidic TME, samples were incubated with 4-(2-hydroxyethyl)-1-piperazineethanesulfonic acid (HEPES) buffer at different pH values (7.4, 6.0, and 5.0) before DLS measurements. In addition, the responsiveness of CLip-PC@CO-LC NPs to the enzyme was also evaluated by digestion with MMP-9 before DLS measurements. The morphologies of the CO-LC NPs, CLip, and CLip-PC@CO-LC NPs, which were diluted with PBS (pH 7.4) and negatively stained with phosphotungstic acid solution (1%, w/v), were observed using TEM (Tecnai G2 S-TWIN, FEI, Hillsboro, OR, USA). To monitor the influence of the acidic environment on the morphology of CLip-PC@CO-LC NPs, the samples were incubated with PBS buffer (pH 5.0) for 1 h before TEM measurements.

### Protein marker characterization

Western blotting assay was conducted to analyze the specific protein markers. Briefly, A549 membrane vesicles (Cell), A549 Cm, CLip, and CLip-PC@CO-LC NPs samples with equivalent protein amounts (40 μg/well) were loaded onto a 10% SDS-PAGE gel, followed by electrophoresis at 85 V for 30 min and 110 V for 80 min. After electroblotting onto polyvinylidene difluoride membranes, the primary anti-histone H3, anti-pan-cadherin, and anti-COX IV were added, followed by incubation at 4 °C overnight and incubation with horseradish peroxidase (HRP)-conjugated secondary antibodies (1:5,000) at RT for 1 h. Na^+^/K^+^-ATPase (Cell Signaling Technology, 3010S, 1:1000) served as an internal control. Films were further visualized using enhanced chemiluminescence (ECL) reagent, and the chemiluminescence signal was imaged using a Bio-imager (Kodak).

### Fusogenic property of CLip-PC@CO-LC NPs

The membrane protein composition of CLip-PC@CO-LC NPs was characterized using SDS-PAGE. Briefly, the Cm, CLipm (CLip membrane), and CLip-PC@CO-LC NPs were lysed using a radioimmunoprecipitation assay (RIPA) lysis buffer to collect proteins and heated at 95 °C for 5 min. Proteins were then loaded on a 10% SDS-PAGE gel (Beyotime, China) and run at 120 V for 1 h, followed by staining with Coomassie Blue and imaging. Subsequently, lipophilic red membrane dye DiD (excitation/emission: 644 nm/665 nm) and green membrane dye DiO (excitation/emission: 484 nm/501 nm) were used to dye the Cm and Lipm, respectively. C-PC@CO-LC NPs, Lip-PC@CO-LC NPs, and CLip-PC@CO-LC NPs were fabricated using the dye-labeled membranes and visualized by using CLSM (Nikon, Tokyo, Japan).

### Agarose gel electrophoresis

According to a previous report [29], the capacity of LC to condense siRNA can be observed using gel retardation assay, and siRNA is completely retarded at an N/P ratio of 4. To investigate the kinetics of siRNA release in the acidic and reductive TME, the kinetics of siRNA release from CLip-PC@CO-LC NPs (N/P = 4) was observed in the presence or absence of DTT at different pH values (7.4, 6.0, or 5.0) for 1 h. Electrophoresis was performed in Tris-acetate-EDTA buffer on 1% (w/v) agarose gels containing 0.01% GelRed (Biotium, Hayward, CA, USA) and run at 100 V for 30 min. The gel was visualized under UV illumination and photographed.

### Evaluation of DTX Release from CLip-PC@CO-LC NPs in vitro

The release profiles of DTX from CLip-PC@CO-LC NPs (with or without 50 mM MMP-9) at different pH values (7.4, 6.0, or 5.0) were evaluated using the dialysis method as described in previous reports [68]. Briefly, 1 mL of CLip-PC@CO-LC NP solution was added to a dialysis bag (MWCO 3500) that was soaked in the release medium (PBS buffer; pH = 7.4, 6.5, and 5.0; 0.1 M) with or without 50 mM MMP-9. Drug release analysis was performed in a water bath at 37 °C with gentle shaking at 100 rpm. At preset testing periods, 0.1 mL aliquots were sampled, and the same volume of fresh medium was added to maintain a consistent volume. The released drug content was quantified by HPLC.

### Cellular uptake assay

Flow cytometry analysis was conducted to further assess the cellular uptake of CLip-PC@CO-LC NPs. CLip-PC@LC-Nile red NPs were fabricated by substituting DTX with Nile Red. Briefly, A549 cells were inoculated in 12-well plates (3 × 10^5^ cells per well) and grown for 24 h at 37 °C in 5% CO_2_. Nile Red, LC-Nile Red, or CLip-PC@LC-Nile red (with or without 50 nM MMP-9) in 100 µL HEPES was added and incubated at 37 °C for 4 h. To investigate the cellular uptake of siPGAM1 mediated by CLip-PC@LC-siPGAM1 NPs, siPGAM1 was replaced with FAM-labeled siPGAM1 (FAM-siPGAM1, 1 × 10^−6^ M). LC was co-incubated with FAM-siPGAM1 at an N/P ratio of 4 for 30 min for further preparation of CLip-PC@LC-siPGAM1 NPs. Then, FAM-siPGAM1, LC-FAM-siPGAM1, and CLip-PC@LC-FAM-siPGAM1 (with or without 50 nM MMP-9) were freshly prepared and incubated with A549 cells in a serum-free medium for 4 h at 37 °C. The positively stained cells and fluorescence intensities were evaluated using flow cytometry. Briefly, after incubation, the cells were trypsinized, harvested, washed with PBS, and suspended. Cellular uptake of FAM-siPGAM1 via CLip-PC@LC-siPGAM1 NPs and CLip-PC@LC-Nile red NPs was evaluated using a flow cytometer (FACSCalibur; BD Biosciences, UK). The experiments were repeated three times.

For CLSM, A549 cells were inoculated on chambered coverslips (5 × 10^4^ cells per well) and cultured overnight. Free Nile Red, LC-Nile Red, CLip-PC@LC-Nile Red, LC-FAM-siPGAM1, CLip-PC@LC-FAM-siPGAM1 (with or without 50 nM MMP-9), or CLip-PC@CO-LC (with and without 50 nM MMP-9, 1 × 10^−6^ M of FAM-siPGAM1, N/P = 4:1) or added into each well. After 4 h of incubation, the cells were washed, fixed with fresh 4% paraformaldehyde, and dyed with DAPI. The cells were then visualized using CLSM (Nikon, Japan).

### Intracellular lysosome escape assay

The lysosomal escape of CLip-PC@LC-FAM-siRNA (with 50 nM MMP-9) in A549 cells was observed by CLSM. Briefly, A549 cells were inoculated in 24-well plates (5 × 10^4^ cells/well) and cultured overnight. Fresh medium containing CLip-PC@LC-FAM-siRNA (with 50 nM MMP-9) was added. After 2 and 4 h of incubation, the cells were incubated with LysoTracker®Red at 37 °C for 1 h. Cells were then stained with DAPI, washed, and fixed in 4% paraformaldehyde. Finally, the samples were visualized using CLSM.

### In vitro cytotoxicity and biocompatibility evaluation

A549 cells were inoculated into 96-well plates at a density of 5 × 10^3^ cells/well and incubated in 5% CO_2_ at 37 °C overnight. The culture medium of each well was refreshed with culture medium containing free DTX, CLip-PC@LC-DTX NPs, CLip-PC@LC-siPGAM1 NPs (DTX: 1 μg/mL; siPGAM1 20 nM), CLip-PC@CO-LC NPs, CLip-PC@CO-LC NPs (with MMP-9), or different concentrations of CLip-PC@Blank-LC (0–150 μg/mL) and incubated for 24 and 48 h. The medium was replaced with 100 μL fresh complete medium containing 10% (v/v) CCK-8 solution and incubated for 2 h in 5% CO_2_ at 37 °C. Absorbance was measured at 450 nm using a plate reader. Cell viability was assayed by comparing the optical density of the treated cells with that of the untreated control.

### Cell cycle and apoptosis assays

A549 cells were seeded in 12-well plates (6 × 10^4^ cells/well), cultured at 37 °C overnight, treated with refreshed culture medium containing CLip-PC@Blank-LC NPs, free DTX, siPGAM1, CLip-PC@LC-DTX NPs, CLip-PC@LC-siPGAM1 NPs, CLip-PC@CO-LC NPs, or CLip-PC@CO-LC NPs (with MMP-9) (DTX: 1 μg/mL; siPGAM1: 20 nM), and incubated for 48 h. After the corresponding time, the cells were collected, washed, fixed with ethanol 70%, and stored overnight at 4 °C. Subsequently, the cells were fixed, washed with PBS, and resuspended in PBS (300 µL), and then RNase A (10 µg/mL) was added. After incubation for 30 min, cells were treated with the DNA intercalating dye PI (10 µg/mL) in the dark for 15 min. Finally, the cell cycle distributions were detected using a FACSCalibur instrument (BD Biosciences, UK) and analyzed using ModiFit software (Topsham, ME, USA). The experiments were conducted in triplicate.

For the apoptosis analysis, the cells were seeded and incubated with the above-mentioned sample groups for 48 h. Subsequently, the cells were trypsinized, collected, washed with PBS, and stained with FITC-Annexin V and PI away from light for 15–30 min; the apoptotic cells were quantified using flow cytometry.

### Measurement of intracellular lactate, ATP level, and glucose uptake

For the detection of intracellular lactate concentration, A549 cells were seeded in a 24-well plate (5 × 10^4^ cells per well) and cultured for 24 h in an incubator (37 °C, 5% CO_2_). CLip-PC@Blank-LC NPs, DTX, siPGAM1, CLip-PC@LC-DTX NPs, CLip-PC@LC-siPGAM1 NPs, CLip-PC@CO-LC NPs, or CLip-PC@CO-LC NPs (MMP-9) were added, and cells were incubated for 6 h. Subsequently, the cells were collected and homogenized. The homogenate was centrifuged at 4 °C for 10 min at 13,000×*g* to remove insoluble material and deproteinized with a 10 kDa MWCO spin filter to remove lactate dehydrogenase. Then, the cells were incubated with the enzyme and substrate working reagent mixture away from light for 30 min at RT. The lactate concentration was determined using lactate assay kit (Sigma-Aldrich, MAK064) by measuring the absorbance at 570 nm according to the manufacturer’s instructions. Each treatment contained three parallel samples.

To measure the intracellular ATP level, A549 cells were inoculated into 24-well plates (5 × 10^4^ cells/well), cultured for 24 h in an incubator (37 °C, 5% CO_2_), and then incubated with CLip-PC@Blank-LC NPs, DTX, siPGAM1, CLip-PC@LC-DTX NPs, CLip-PC@LC-siPGAM1 NPs, CLip-PC@CO-LC NPs, or CLip-PC@CO-LC NP (MMP-9). After incubation for 6 h, cells were collected, and the intracellular ATP levels were measured using the Kinase-Glo® Luminescent Kinase Assay kit (Promega, V6711) following the manufacturer’s instructions. The luminescent signals of ATP were detected by a GloMax®-Multi Jr Single-Tube Multimode Reader. Each sample was replicated in three wells.

The glucose uptake of A549 cells was analyzed using a glucose uptake assay kit (Invitrogen, MA, USA) as previously reported [69]. Briefly, the cells were seeded in a 96-well plate at a density of 1.2 × 10^4^ cells, cultured in a serum-free medium for 24 h, and washed twice with a glucose-free serum-free medium. Subsequently, they were incubated with the above-mentioned sample groups for 6 h. The culture medium was discarded, the cells were washed twice with PBS, 100 μL/well of the 2-NBDG solution was added to each well, and the plate was incubated for 30 min in a 5% CO_2_ incubator at 37 °C. Thereafter, the cells were washed three times with ice-cold PBS (200 μL/well), and 70 μL of 0.1 M potassium phosphate buffer (pH 10) containing 1% Triton X-100 was added to each well. The cells were then kept in the dark for 10 min. Finally, 30 μL of DMSO was added, and the mixture was homogenized by pipetting it into the well. Fluorescence was measured immediately using a microplate reader (λ_ex_ = 465 nm, λ_em_ = 540 nm).

### NADPH/NADP+ and intracellular 2- and 3-PG concentration measurement

The NADPH/NADP^+^ ratio was determined using an EnzyFluo™ NADP/NADPH Assay Kit (BioAssay) according to the manufacturer’s protocol. Cellular metabolites were extracted and spectrophotometrically detected as previously described [15, 70, 71]. To measure the intracellular 2- and 3-PG levels, cells were collected after incubation with CLip-PC@Blank-LC NPs, DTX, siPGAM1, CLip-PC@LC-DTX NPs, CLip-PC@LC-siPGAM1 NPs, CLip-PC@CO-LC NPs, or CLip-PC@CO-LC NPs (MMP-9) for 24 h. Each group of cells was homogenized in 1.5 mL of hypotonic lysis buffer (20 mM HEPES [pH 7.0], 5 mM KCl, 1 mM MgCl_2_, 5 mM DTT, and protease inhibitor cocktail). Subsequently, the homogenates were centrifuged at 4 °C for 10 min at 13,000 rpm, and the supernatants were applied to a 10 kDa MWCO spin filter (Millipore). The flow-through containing the metabolites was used for the measurements.

### Transwell migration and invasion assays

The scratch-wound cell migration assay was performed as previously described [72]. Briefly, A549 cells were inoculated (3 × 10^5^ cells/mL, 70 μL/well) in Ibidi Culture-Inserts (Ibidi, Martinsried, Germany). After 24 h, cell growth was observed in a confluent cell layer and the formation of a well-defined gap. The cells were washed and incubated with serum-free medium containing CLip-PC@Blank-LC NPs, DTX, siPGAM1, CLip-PC@LC-DTX NPs, CLip-PC@LC-siPGAM1 NPs, CLip-PC@CO-LC NPs, or CLip-PC@CO-LC NPs (MMP-9) (DTX: 1 μg/mL; siPGAM1: 20 nM). The gap closure was photographed at 0, 24, and 48 h under a Leica microscope (Leica Microsystems, Wetzlar, Germany).

Matrigel invasion analysis was conducted in Matrigel invasion chambers (BD Biosciences, Bedford, MA, USA). Briefly, A549 cells were pre-treated with CLip-PC@Blank-LC NPs, DTX, siPGAM1, CLip-PC@LC-DTX NPs, CLip-PC@LC-siPGAM1 NPs, CLip-PC@CO-LC NPs, or CLip-PC@CO-LC NPs (MMP-9) (DTX: 1 μg/mL; siPGAM1: 20 nM) for 48 h. Then, the cells were collected and seeded (1 × 10^4^ cells per chamber) in the Matrigel-coated upper chamber, and a 10% FBS-containing medium was added to the lower chamber. After incubation for 24 h, cells on the upper surface were gently scraped, and the cells that migrated to the lower surface of the Transwell chambers were fixed and stained with 0.1% crystal violet for 30 min. Cells were counted and photographed under a Leica microscope (Leica Microsystems, Wetzlar, Germany).

### Western blotting analysis

The gene silencing effects of CLip-PC@CO-LC NPs were evaluated using Western blotting. Briefly, A549 cells were treated with siPGAM1, DTX, CLip-PC@LC-DTX NPs, CLip-PC@LC-siPGAM1 NPs, or CLip-PC@CO-LC NPs (DTX: 1 μg/mL; siPGAM1: 20 nM) for 48 h. Then, all the cells of each group were collected and lysed in 100 μL RIPA lysis buffer containing 1% PMSF. The protein was quantified by BCA assay and boiled with a loading buffer. Equal amounts of protein were separated by SDS-PAGE and transferred onto a polyvinylidene fluoride (PVDF) membrane. After blocking with 5% non-fat milk, the membranes were probed with antibodies against PGAM1 and HRP-conjugated secondary antibodies (1:4000), and the target proteins were visualized using ECL (Millipore, Bedford, MA, USA). β-Actin protein was used as an internal standard.

### Quantitative RT-PCR

The gene-silencing effects of CLip-PC@CO-LC NPs were investigated by quantitative RT-PCR using PGAM1 as a target gene. A549 cells were seeded in a 6-well plate (1 × 10^6^ cells/well) for 24 h. The culture medium was replaced with fresh medium containing siPGAM1, DTX, CLip-PC@LC-DTX NPs, CLip-PC@LC-siPGAM1 NPs, or CLip-PC@CO-LC NPs (DTX: 1 μg/mL; siPGAM1: 20 nM). Then, each group of cells was collected, and the total RNA was extracted using Trizol Reagent (Invitrogen). Subsequently, cDNA was synthesized using a SuperScript II reverse transcriptase kit (Takara Bio, USA). Quantitative RT-PCR was performed using an SYBR Green ER quantitative PCR SuperMix Universal kit (Thermo Fisher Scientific, USA). Reactions were carried out using a standard cycle program on an AB7500 real-time PCR system. The sequences of the forward and reverse primers of PGAM1 were 5′-GCACCCACTCCCTTCATACAAT and 5′-ACGCAGGTTACATTCGTCTTCC, respectively. The sequences of the forward and reverse primers for β-actin were 5′-ACAGAGCCTCGCCTTTGC and 5′-AGAGGCGTACAGGGATAGCA, respectively. The RT-PCR reaction parameters were as follows: 95 °C for 10 min; 95 °C for 15 s followed by 60 °C for 1 min, for 40 cycles. The relative gene expression was quantified using the ^ΔΔ^Ct method.

### Biodistribution

To investigate the biodistribution, 1 × 10^7^ A549 cells (100 μL) were injected subcutaneously into the right flanks of BALB/c nude mice (18–20 g). When the average tumor size reached 50 mm^3^, free DiR or CLip-PC@LC-DiR NPs (2 mg/kg DiR, n = 5 per group) were infused into the mice through the caudal vein. A noninvasive optical in vivo imaging system was used to collected fluorescence images at 1, 3, 6, 12, and 24 h after administration. At 24 h after injection, the mice were sacrificed, and tumors and major organs (heart, liver, spleen, lung, and kidney) were collected for fluorescence imaging.

### Antitumor effect in vivo

To study the tumor-inhibiting efficacy of CLip-PC@CO-LC NPs in vivo, an A549 cell tumor-bearing mouse model was established by subcutaneous injection of A549 cells in the right flank region of each mouse. When the average tumor size reached 50 mm^3^, the mice were randomly divided into seven groups (n = 6): (1) saline; (2) CLip-PC@Blank-LC; (3) DTX; (4) siPGAM1; (5) CLip-PC@LC-DTX NPs; (6) CLip-PC@LC-siPGAM1 NPs; and (7) CLip-PC@CO-LC NPs (DTX: 10 mg/kg, siPGAM1: 2 mg/kg). During the therapeutic process, the tumor size and mouse weight were recorded every 3 days, and the tumor size (V) was calculated as V = (length × width)^2^/2. All mice were sacrificed on day 20, and the tumors were collected, weighed, and fixed in 4% paraformaldehyde for histological sectioning. The survival of the mice after injection was also recorded. Tumor growth inhibition (TGI%) was calculated as:

$${\text{TGI}}\% = 1 - \left( {{\text{Wt}} - {\text{Wi}}} \right)/{\text{Ws}} - {\text{Wi}}) \times 100\% ,$$

where Wt is the final average tumor weight of the tested groups, Wi is the initial average tumor weight of each test subject-treated group, and Ws is the final average tumor weight of the saline group.

### Histology and IHC analysis

After the mice were euthanized, the tumors and major organs were extracted for HE staining, which was used to evaluate tissue damage. To investigate the in vivo gene-silencing effects, the expression of PGAM1 in tumors was examined using IHC. TUNEL staining was performed to investigate apoptotic cells in tumor slices. Ki67 IHC was performed using standard protocols supplied by the Human Ki67 Detection Kit (DAKO, Glostrup, Denmark). TUNEL-positive (apoptotic) or Ki67-positive (proliferating) cells were stained dark brown, and nuclei were counterstained with hematoxylin. Photographs of the sections were obtained using an inverted microscope (Olympus, Tokyo, Japan) and the Aperio ScanScope Image Analysis System (Aperio, Vista, CA, USA).

### Statistical analysis

All results are expressed as the mean ± standard error. Inter-group differences were tested using one-way analysis of variance (ANOVA), and p-values less than 0.05 indicated significant differences.

## Supplementary Information

**Additional file 1: Figure S1.** ^1^H NMR spectra of PC. **Figure S2.** Characterization of CLip-PC@CO-LC NPs. Nanoparticle size distribution (a) and Zeta potential (b) of CLip-PC@CO-LC NPs at pH 7.4, 6.0, and 5.0, determined by DLS. Hydrodynamic diameter (c) and zeta potential (d) of CLip-PC@CO-LC NPs treated with or without MMP-9. **Figure S3.** The biocompatibility of CLip-PC@Blank-LC NPs. The cells were treated with Clip-PC@Blank-LC NPs during 0–150 μg/mL for 24 h and 48 h, respectively.

## Data Availability

All data generated or analyzed during this study are included in this published article.

## Abbreviations

PGAM1: Phosphoglycerate mutase 1
3-PG: 3-Phosphoglycerate
2-PG: 2-Phosphoglycerate
CPP: Cell-penetrating peptide
Cm: Cancer cell membrane
Lipm: MMP-9-switchable peptide-based charge-reversal liposome membrane
TME: Tumor microenvironment
CLip: Hybrid membrane prepared by integrating cancer cell membrane and MMP-9-switchable peptide-based charge-reversal liposome membrane
PC: Citraconic anhydride-grafted poly-l-lysine
FRET: Förster resonance energy transfer
FTIR: Fourier transform infrared
DLS: Dynamic light scattering
ECL: Enhanced chemiluminescence
siPGAM1: *PGAM1* SiRNA
LC: Lipoic acid-modified polypeptide
SDS-PAGE: Sodium dodecyl sulfate-polyacrylamide gel electrophoresis
RIPA: Radioimmunoprecipitation assay
CLSM: Confocal laser scanning microscopy
TAE: Tris-acetate-EDTA
PI: Propidium iodide
PMSF: Phenylmethanesulfonyl fluoride
BCA: Bicinchoninic acid
IVIS: In vivo imaging system
H&E: Hematoxylin and eosin
TUNEL: TdT-mediated dUTP Nick-End Labeling
PDI: Polymer dispersity index
DiD: 1,1′-Dioctadecyl-3,3,3′,3-tetramethylindodicarbocyanine, 4-chlorobenzenesulfonate salt
DiO: 3,3-Dioctadecyloxacarbocyanine perchlorate
DTT: Dithiothreitol
PPP: Phosphopentose pathway
