# Supplementary material for: Tumor microenvironment-activated cancer cell membrane-liposome hybrid nanoparticle-mediated synergistic metabolic therapy and chemotherapy for non-small cell lung cancer
Source: J Nanobiotechnology. 2021 Oct 24;19:339. doi: 10.1186/s12951-021-01085-y (PMC8543810; doi:10.1186/s12951-021-01085-y)
Supplement: Supplementary file 1 — Additional file 1: Figure S1. 1H NMR spectra of PC. Figure S2. Characterization of CLip-PC@CO-LC NPs. Nanoparticle size distribution (a) and Zeta potential (b) of CLip-PC@CO-LC NPs at pH 7.4, 6.0, and 5.0, determined by DLS. Hydrodynamic diameter (c) and zeta potential (d) of CLip-PC@CO-LC NPs treated with or without MMP-9. Figure S3. The biocompatibility of CLip-PC@Blank-LC NPs. The cells were treated with Clip-PC@Blank-LC NPs during 0–150 μg/mL for 24 h and 48 h, respectively. [file 12951_2021_1085_MOESM1_ESM.docx]

**Additional files**

Tumor Microenvironment-Activated Cancer Cell Membrane-Liposome Hybrid Nanoparticle-Mediated Synergistic Metabolic Therapy and Chemotherapy for Non-Small Cell Lung Cancer

Wei Zhang^a, #^, Chunai Gong^b, #^, Ziqiang Chen^c, #^, Ming Li^c^, Yuping Li ^a, *^, Jing Gao^d, *^

1. **Synthesis of citraconic anhydride grafted poly-L-lysine (PC)**

**
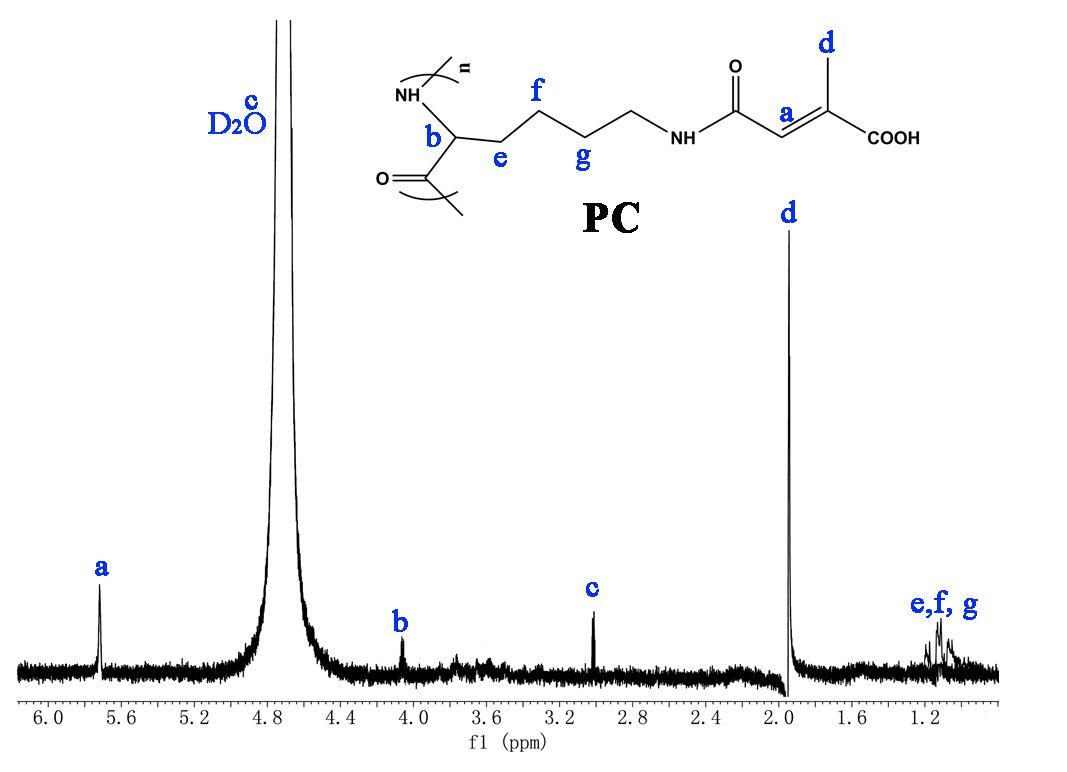
**

Figure S1. ^1^H NMR spectra of PC

**2. Characterization of CLip-PC@CO-LC NPs**

**
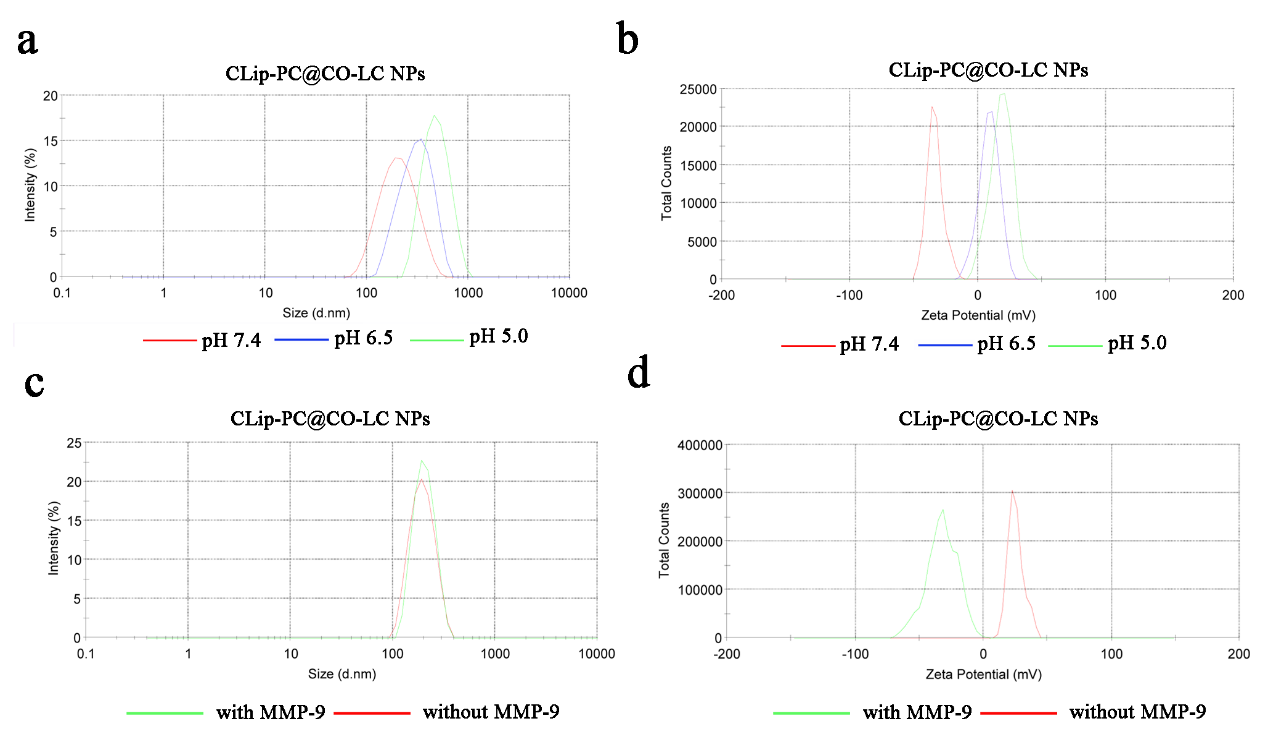
**

Figure S2. Characterization of CLip-PC@CO-LC NPs. Nanoparticle size distribution a) and Zeta potential b) of CLip-PC@CO-LC NPs at pH 7.4, 6.0, and 5.0, determined by DLS. Hydrodynamic diameter (c) and zeta potential (d) of CLip-PC@CO-LC NPs treated with or without MMP-9.

**3. *In vitro* Biocompatibility evaluation**


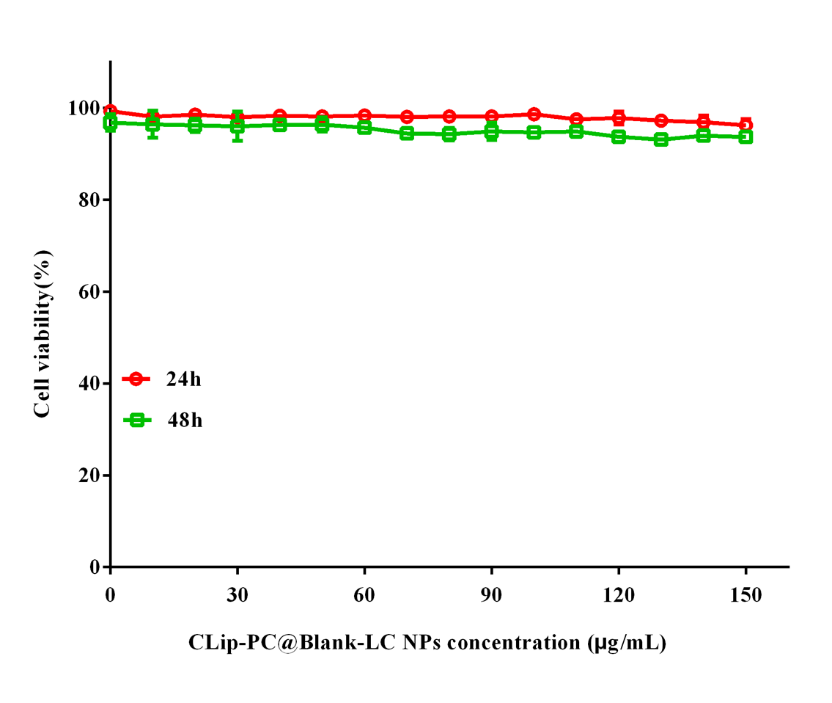


Figure S3. The biocompatibility of CLip-PC@Blank-LC NPs. The cells were treated with Clip-PC@Blank-LC NPs during 0–150 μg/mL for 24 h and 48 h, respectively.
